# Gut microbiota functional profiling in autism spectrum disorders: bacterial VOCs and related metabolic pathways acting as disease biomarkers and predictors

**DOI:** 10.3389/fmicb.2023.1287350

**Published:** 2023-12-18

**Authors:** Pamela Vernocchi, Chiara Marangelo, Silvia Guerrera, Federica Del Chierico, Valerio Guarrasi, Simone Gardini, Federica Conte, Paola Paci, Gianluca Ianiro, Antonio Gasbarrini, Stefano Vicari, Lorenza Putignani

**Affiliations:** ^1^Research Unit of Human Microbiome, Bambino Gesù Children’s Hospital, IRCCS, Rome, Italy; ^2^Child and Adolescent Neuropsychiatry Unit, Department of Neuroscience, Bambino Gesù Children's Hospital, IRCCS, Rome, Italy; ^3^GenomeUp, Rome, Italy; ^4^Institute for Systems Analysis and Computer Science “Antonio Ruberti”, National Research Council, Rome, Italy; ^5^Department of Computer, Control and Management Engineering, Sapienza University of Rome, Rome, Italy; ^6^CEMAD Digestive Disease Center, Fondazione Policlinico Universitario "A. Gemelli" IRCCS, Università Cattolica del Sacro Cuore, Rome, Italy; ^7^Life Sciences and Public Health Department, Università Cattolica del Sacro Cuore, Rome, Italy; ^8^Unit of Microbiomics and Research Unit of Human Microbiome, Bambino Gesù Children’s Hospital, IRCCS, Rome, Italy

**Keywords:** autism, gut microbiota volatilome, tryptophan-derived metabolism, SCFAs, machine learning, clinical decision support system (CDSS) algorithms

## Abstract

**Background:**

Autism spectrum disorder (ASD) is a multifactorial neurodevelopmental disorder. Major interplays between the gastrointestinal (GI) tract and the central nervous system (CNS) seem to be driven by gut microbiota (GM). Herein, we provide a GM functional characterization, based on GM metabolomics, mapping of bacterial biochemical pathways, and anamnestic, clinical, and nutritional patient metadata.

**Methods:**

Fecal samples collected from children with ASD and neurotypical children were analyzed by gas-chromatography mass spectrometry coupled with solid phase microextraction (GC–MS/SPME) to determine volatile organic compounds (VOCs) associated with the metataxonomic approach by 16S rRNA gene sequencing. Multivariate and univariate statistical analyses assessed differential VOC profiles and relationships with ASD anamnestic and clinical features for biomarker discovery. Multiple web-based and machine learning (ML) models identified metabolic predictors of disease and network analyses correlated GM ecological and metabolic patterns.

**Results:**

The GM core volatilome for all ASD patients was characterized by a high concentration of 1-pentanol, 1-butanol, phenyl ethyl alcohol; benzeneacetaldehyde, octadecanal, tetradecanal; methyl isobutyl ketone, 2-hexanone, acetone; acetic, propanoic, 3-methyl-butanoic and 2-methyl-propanoic acids; indole and skatole; and o-cymene. Patients were stratified based on age, GI symptoms, and ASD severity symptoms. Disease risk prediction allowed us to associate butanoic acid with subjects older than 5 years, indole with the absence of GI symptoms and low disease severity, propanoic acid with the ASD risk group, and p-cymene with ASD symptoms, all based on the predictive CBCL-EXT scale. The HistGradientBoostingClassifier model classified ASD patients vs. CTRLs by an accuracy of 89%, based on methyl isobutyl ketone, benzeneacetaldehyde, phenyl ethyl alcohol, ethanol, butanoic acid, octadecane, acetic acid, skatole, and tetradecanal features. LogisticRegression models corroborated methyl isobutyl ketone, benzeneacetaldehyde, phenyl ethyl alcohol, skatole, and acetic acid as ASD predictors.

**Conclusion:**

Our results will aid the development of advanced clinical decision support systems (CDSSs), assisted by ML models, for advanced ASD-personalized medicine, based on omics data integrated into electronic health/medical records. Furthermore, new ASD screening strategies based on GM-related predictors could be used to improve ASD risk assessment by uncovering novel ASD onset and risk predictors.

## 1 Introduction

Autism spectrum disorder (ASD) is a widespread neurodevelopmental condition characterized by neurological disorders, including impairments in social communication, reciprocity, and repetitive behavior patterns, frequently associated with recurrent comorbidities such as gastrointestinal (GI) disorders (Mottron and Bzdok, 2020), sleep disturbances, and epilepsy (Chen et al., 2021; Madra et al., 2021; Liu et al., 2022). Particularly, most patients affected by ASD, generally males (Loomes et al., 2017), show GI morbidities (Mottron and Bzdok, 2020), often associated with impaired digestion of carbohydrates and gut dysbiosis (Williams et al., 2011), hence altered gut microbiota (GM) composition and metabolism may play a crucial role in ASD phenotypes and comorbidities (De Angelis et al., 2015; Vernocchi et al., 2022). GM microbial profiles (Shoubridge et al., 2022) have been thoroughly investigated in terms of gut microbial ecology in ASD children compared with neurotypical children (De Angelis et al., 2015; Adams et al., 2022; Nirmalkar et al., 2022, 2023; Phan et al., 2022; Taniya et al., 2022; Vernocchi et al., 2022). However, through the “gut-brain axis,” gut microbial-driven metabolites (Järbrink-Sehgal and Andreasson, 2020) may exert crucial effects on the physiology of the central nervous system (CNS) and association to ASD (Thomas et al., 2012; MacFabe, 2015).

Indeed, gut metabolic dysfunction can be associated with GI symptoms, such as deficits in digestion/absorption, or, conversely, GI tract alterations may impact microbial community eubiosis, contributing to enhancing only specific bacterial metabolites, and thereby triggering pro-inflammatory responses, cytokine production, and loss of gut epithelial barrier integrity (Martin et al., 2018). Therefore, a bottom-up CNS modulation, exerted by GM-derived molecules, mainly occurs through neuroimmune and neuroendocrine pathways, typically through the vagus nerve (Singh et al., 2016; Hughes et al., 2018) via chemoreceptors and mechanoreceptors stimulated by microbial metabolites or bacterial taxa, respectively (Ristori et al., 2019). Amongst bacterial molecules, short-chain fatty acids (SCFAs) and tryptophan-derived metabolites (i.e., indoles, skatole) (Yano et al., 2015; Hughes et al., 2018) play an important role in the GM-brain interplay (Li et al., 2021).

This study explored the GM volatilome to assess the most significant gut metabolic perturbations depending on ASD phenotype and comorbidities and to identify novel disease biomarkers and predictors.

## 2 Materials and methods

### 2.1 Patient enrolment and sample collection

This observational cohort study was conducted in Italy at the Bambino Gesù Children’s Hospital (OPBG) Rome, Italy. For the study, 41 ASD patients aged 3–15 years (36 boys and five girls) were recruited at OPBG and Agostino Gemelli Hospital in Rome, Italy, with a diagnosis of ASD based on the criteria of the Diagnostic and Statistical Manual of Mental Disorders DSM-5 and confirmed by the Autism Diagnostic Observation Schedule (ADOS-2) and by the Autism Diagnostic Interview – Revised (ADI-R). ASD patients were aged-matched against a cohort of 35 neurotypical children (21 boys and 14 girls) (controls, CTRLs) selected during a GM programming survey at the OPBG Human Microbiome Unit (Vernocchi et al., 2022).

The anamnestic and clinical data collected during this study included gender; age; weight; height; BMI; type of birth; infant feeding; GI symptoms categorized into presence and absence subsets and reported according to Rome IV criteria (Drossman and Hasler, 2016) (i.e., constipation, diarrhea, abdominal distention, abdominal pain, gastroesophageal reflux disease (GERD), colic or eosinophilia); neuropsychological features reported as autism symptom severity (i.e., low, moderate, high); behavioral problems, reported as presence of risk for behavioral problems; absence of clinical symptoms based on CBCL (Child Behavior Checklist)-INT, CBCL-EXT, and CBCL-TOT scales; cognitive level reported as IQ/DQ (Intelligence Quotient/Developmental Quotient) with and without cognitive impairment or developmental delay; pharmacological treatment; and epilepsy. Nutritional habits, at the time of subject recruitment, were defined in terms of disposition to be a Picky Eater (PE) or not, namely subjects with food selectivity (FS) and/or with a gluten-free or casein-free diet (Supplementary Table S1; Vernocchi et al., 2022).

GM metabolomic profiling was performed on fecal samples from each subject. Samples were taken at home and refrigerated and delivered during clinical visits. The fecal samples were stored on ice ore at +4°C and transferred to the laboratory within 2 h. All samples were collected by the same researcher using standardized procedures. The freezing tube did not include preservatives. In total, 76 fecal samples were collected and stored at −80°C at the Microbiome Biobank of the OPBG, node of the Biobanking and Biomolecular Resources Research Infrastructure of Italy (BBMRI) of the Human Microbiome Unit until processing for GM metataxonomy (Vernocchi et al., 2022) and metabolomics.

### 2.2 Ethics statement

The study was approved by the OPBG Ethics Committee for both patients with ASD (1404_OPBG_2017) and healthy subject (1113_OPBG_2016) cohorts and conducted in accordance with the Principles of Good Clinical Practice and the Declaration of Helsinki. Written informed consent was obtained from all participants.

### 2.3 GM metataxonomy

GM taxonomic profiles were built as previously described (Vernocchi et al., 2022). Specifically, the extracted DNA from 41 and 35 fecal samples of ASD and CTRLs subjects, respectively, was performed using a QIAmp Fast DNA Stool mini kit (Qiagen, Hilden, Germany) consistent with the manufacturer’s instructions.

Amplification of the variable region V3–V4 from the bacterial 16S rRNA gene (∼460 bp) and the PCR reaction were set up according to Vernocchi et al. (2022). The final libraries were cleaned up using CleanNGS kit beads, quantified with Quant-iT PicoGreen dsDNA Assay Kit (Thermo Fisher Scientific, Waltham, MA, United States), and normalized to 4 nM. To produce paired-end 250- × −2 bp-length reads, normalized libraries were united together and run on the Illumina MiSeq platform according to specifications of the manufacturer. The obtained raw reads were processed using Quantitative Insights into Microbial Ecology software (QIIME, 1.9.1) (Caporaso et al., 2010), according to Vernocchi et al. (2022).

### 2.4 GM metabolomic profiling by determination of volatile organic compounds (VOCs)

#### 2.4.1 Gas chromatography-mass spectrometry

Fecal samples from 41 patients and 35 CTRLs were analyzed by GC–MS to detect VOCs by using a carboxen-polydimethylsiloxane coated fiber (85 μm) and a manual solid-phase micro-extraction holder (Supelco Inc., Bellefonte, PA, United States) as previously described (Vernocchi et al., 2020).

### 2.5 Biocomputational approaches: multivariate, univariate, and correlation analyses

Metabolite datasets were filtered based on the criterion of metabolite presence in at least 10% of the total sample set, referring to both ASDs and CTRLs. β-diversity of ASD vs. CTRLs, with ASD subgroups (≤5 years old vs. >5 years old; gender; with vs. without GI symptoms; ASD high vs. low symptoms; disposition to be PE vs. not to be PE), were based on the Bray–Curtis dissimilarity algorithm (Chen et al., 2012) and represented by Principal coordinate analyses (PCoA). Tests were performed by using Python 3.8 version with Skbio.diversity package.

An explorative multivariate (Principal Component Analysis, PCA) analysis was applied to ASDs vs. CTRLs, whilst a partial least square-discriminant analysis (PLS-DA) (Bylesjö et al., 2007) was exploited (Li et al., 2023) by mixOmics R package to identify differential VOCs profiles. Results were verified by univariate analysis with Mann–Whitney test and *p* values adjusted by FDR (Benjamini and Hochberg, 1995). Fold change (FC) was computed as the ratio of average VOCs concentration values for ASDs and CTRLs mean [VOCs_(ASDs)_]/mean [VOCs_(CTRLs)_].

Z-score-based heat maps of VOCs’ distribution were obtained with a hierarchical Ward linkage clustering based on Euclidean distance, by considering only statistically significant variables filtered by Wilcoxon Mann Whitney test (*p* value ≤ 0.05 corrected for multiple hypothesis by Benjamini-Hochberg-based FDR) (Benjamini and Hochberg, 1995), and plotted with R software (Pheatmap package).

Anamnestic features were evaluated as potential confounding factors, while clinical and nutritional features were exploited to stratify the ASD phenotype in terms of symptoms, comorbidities, and nutritional habits and to assess related biomarkers.

The Upset plot was used to visualize intersections of different ASD subgroup combinations and CTRL condition, showing the distribution of uniquely detected metabolites. To generate the Upset plot, the UpSetR R package was applied.

All R-based scripts can be found in the study repository as described in the Data availability section.

### 2.6 Metabolic set enrichment analysis (MSEA) and metabolic pathway analysis (MetPA)

To identify and interpret the metabolic pathways characterizing the GM of ASDs compared to CTRLs, MSEA and MetPA computations were applied (Xia and Wishart, 2010). Metabolites were identified based on chemical names, and annotations were verified using the Human Metabolome Database (HMDB), Kyoto Encyclopedia of Genes and Genomes (KEGG), Small Molecular Pathway Database (SMPDB), PubChem, chemical entities of biological interest (ChEBI), and METLIN databases. These analyses were performed using the MetaboAnalyst (version 4.0) platform.

### 2.7 Network correlation analysis on operational taxonomic units (OTUs) and VOCs

A correlation network between 16S rRNA sequencing-based OTUs (Vernocchi et al., 2020) and VOCs, filtered as statistically significant after a multiple non-parametric Mann–Whitney U-test, was established for ASDs and CTRLs. In particular, we applied a hard thresholding approach which creates binary networks where significant inter-node correlations (*p* value adjusted ≤0.05) are retained (edge values set to 1), whereas no significant correlations (*p* value adjusted >0.05) are removed (edge values set to 0). The network was performed by psych R package and visualized with Cytoscape v3.8.2.

### 2.8 Weighted gene co-expression network analysis (WGCNA) on clinical and anamnestic data, OTUs, and VOCs

WGCNA (Zhang and Horvath, 2005) was performed on 41 ASDs and 35 CTRLs considering the following datasets: (i) VOCs obtained with a 10% cut-off (i.e., non-zero values in at least 10% of the total samples’ number); (ii) OTUs from three taxonomic levels (Level L2, Phylum; Level L5, Family; Level L6, Genus) with a 25% cut-off (i.e., non-zero values in at least 25% of the total samples’ number), and a filter based on the OTUs relative abundance considering those with relative abundance >1%; and (iii) OTUs, VOCs, and clinical and anamnestic features. The VOC and OTU data were combined and log-transformed prior to the downstream statistical analyses (Langfelder and Horvath, 2008). The complete data matrix included 110 VOCs and 161 OTUs and the R package WGCNA (Langfelder and Horvath, 2008) was used to build a weighted correlation network where edge weights were given by a continuous mapping of correlation values (soft thresholding approach). In addition, WGCNA enabled the identification of VOC and OTU network modules with a co-expression similarity (Langfelder and Horvath, 2008) and the incorporation of the external sample traits (i.e., clinical and anamnestic data) in order to screen for modules and intramodular features that were strongly associated with specific traits of interest. Specifically, WGCNA produced a set of modules (labeled by color), each containing a set of unique nodes (OTUs and VOCs). To summarize the information contained in a given module of the WGCNA network, the module eigengene (ME), defined as the PC1 of a given module, was used (Langfelder and Horvath, 2008). Further, for each node in a given module, the module membership (MM) and the biomarker significance (BS) were computed (Langfelder and Horvath, 2008). The MM of a node represents the correlation between the node profile and the ME, whereas the BS of a node represents the correlation between the node profile and a given anamnestic or clinical feature. If the MM of a node is close to 0, the node is not considered a part of the module. Conversely, if the MM is 1 or − 1, the node is highly representative of the module. The sign of the MM explains if the node has a positive or a negative relationship with the ME. Concerning the BS, the greater the absolute value of the BS of a node, the more biologically significant the node is. A BS of 0 indicates that the node is not significant with reference to the feature of interest. The WGCNA R package can be downloaded from CRAN repository.

### 2.9 Multiple machine learning (ML) models

Multiple ML models (i.e., DummyClassifier, KNeighborsClassifier, LogisticRegression CV, LinearSVC, SVC, LogisticRegression, GradientBoostingClassifier, GaussianProcessClassifier, QuadraticDiscriminantAnalysis, SGDClassifier, RandomForestClassifier, BaggingClassifier, DecisionTreeClassifier, GaussianNB, ExtraTreesClassifier, AdaBoostClassifier, MLPClassifier, and HistGradientBoostingClassifier) were trained for searching ranking tasks of ASD vs. CTRL metabolomic profiles. To evaluate the predictive accuracy of the top-ranking models, the performance was assessed by model score. The machine learning model was built with Python (3.8 version) by using scikit-learn package (1.3.1 version). Accuracy, sensitivity, and specificity and odds ratios were computed only for the Logistic Regression model applied to biomarkers discovery from univariate analyses, and receiver operating characteristic (ROC) curves were represented with the relative area under the curve (AUC) values (Wang et al., 2021). The AUC of the model was considered excellent if >0.9, very good if ranging from 0.8 to 0.9, good if associated to the averaged values 0.6–0.8, and poor in the case of <0.6. Predictor evaluations and ROC graphs were performed by ROCR R package.

## 3 Results

### 3.1 Fecal VOCs identification

Overall, 626 VOCs, for both ASDs and CTRLs, were identified by GC–MS/SPME, quantified, and classified into 23 chemical groups: alcohols (*n* = 137), alkenes (*n* = 60), alkanes (*n* = 55), ketones (*n* = 67), esters (*n* = 89), acids (*n* = 31), amides (*n* = 4), phenols (*n* = 12), pyridines (*n* = 2), pyrazines (*n* = 7), indoles (*n* = 11), aldehydes (*n* = 47), aromatic hydrocarbons (*n* = 15), furans (*n* = 3), furfural (*n* = 1), terpenes (*n* = 46), sulfur compounds (*n* = 3), amines (*n* = 24), thiols (*n* = 3), piperazines (*n* = 2), furanones (*n* = 4), hydrazine (*n* = 2), and other organic compounds (*n* = 1).

All data comparisons were performed on a reduced matrix of 110 out of 626 total VOCs (Supplementary Table S2), according to criteria reported in M&M section.

### 3.2 GM volatilome exploitation: ASDs vs. CTRLs sets

For volatilome exploratory data analysis, the β-diversity algorithm, based on Bray Curtis dissimilarity metrics, showed a statistically significant distance (*p* value = 0.01) in the bacterial communities of ASDs and CTRLs datasets (Supplementary Figure S1A). β-diversity was also performed for ASD subgroups to evaluate confounders affecting GM volatilome distribution, according to (i) age, (ii) gender, (iii) presence and absence of GI symptoms, (iv) autism symptoms severity, and (v) disposition to be PEs or not.

For age (≤5 years [23 ASDs and 11 CTRLs] and > 5 years old [18 ASDs and 24 CTRLs]), Bray Curtis dissimilarity plots, including CTRLs (Supplementary Figure S1B) or not (Supplementary Figure S1C), were characterized by a statistically significant distance (*p* ≤ 0.05). Gender subgroups provided statistically different β-diversity representation (*p* = 0.047) (Supplementary Figure S1D).

For presence (30 ASDs) and absence (11 ASDs) of GI symptoms (Supplementary Figure S2A) and disposition to be PEs (24/41) or not (17/41) (Supplementary Figure S2B), Bray Curtis dissimilarity plots, including CTRLs, were characterized by a statistically significant distance (*p* = 0.001).

**Figure 1:**
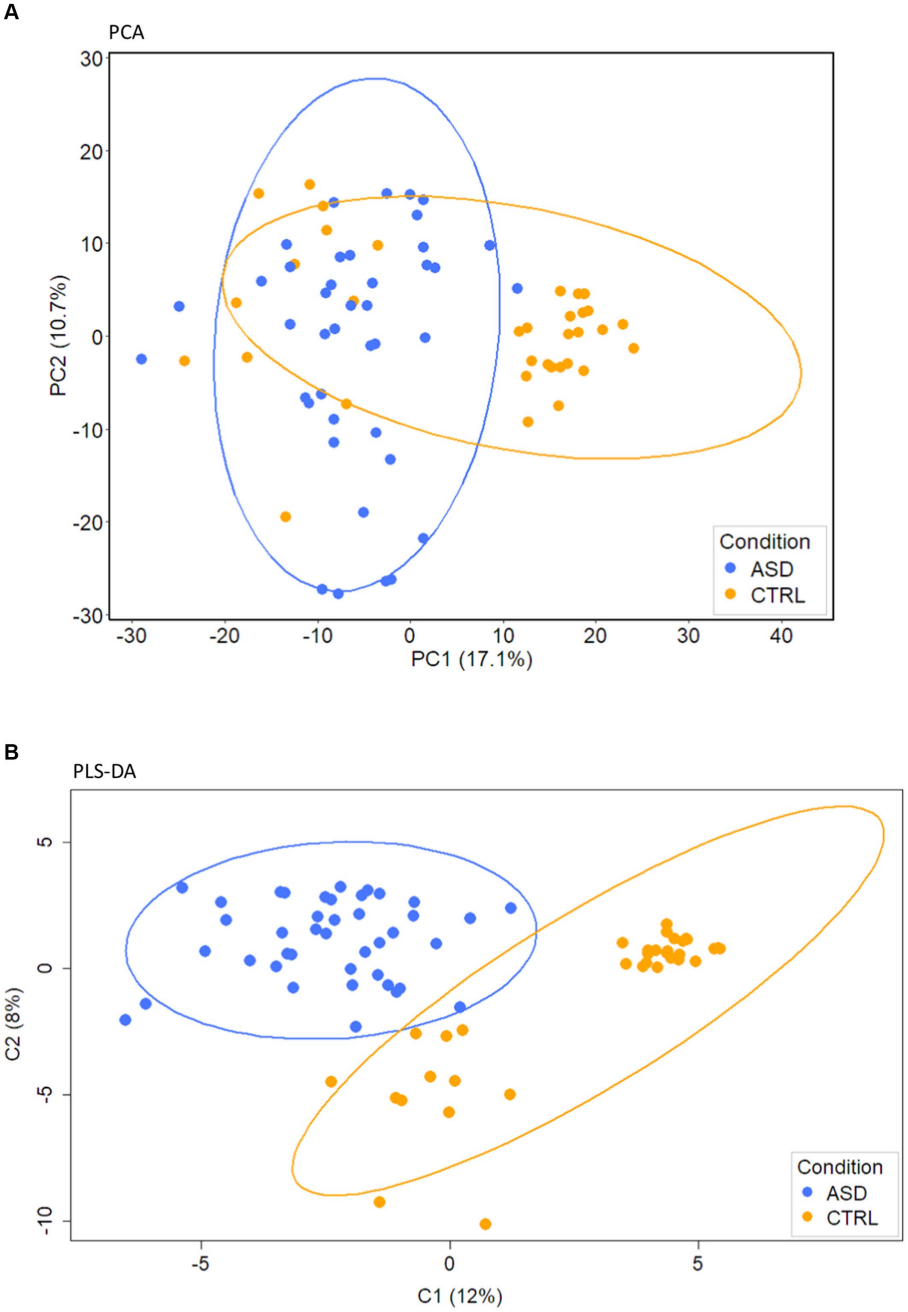
PCA and PLS-DA analyses of gut volatilome. **(A)** PCA. **(B)** PLS-DA score plot of VOCs detected in fecal samples collected from ASD patients and CTRLs.

PCA, performed on the filtered subset of 110 VOCs from 76 samples (41 ASDs and 35 CTRLs), identified two distinct clusters mainly separated along the first two principal components, PC1 and PC2, with a percentage of variances explained of about 29% (Figure 1A; Supplementary Table S3).

A stable PLS-DA (Figure 1B), characterized by 0.15 index of explanation ability and 0.637 of model predictive performance, assessed well the separation of ASDs and CTRLs along C1 and C2 component directions (Figure 1B). PLS-DA loadings of the component C1 (Supplementary Figure S3) were 27 VOCs (Supplementary Table S4) associated with CTRLs and 83 with ASDs.

**Figure 2:**
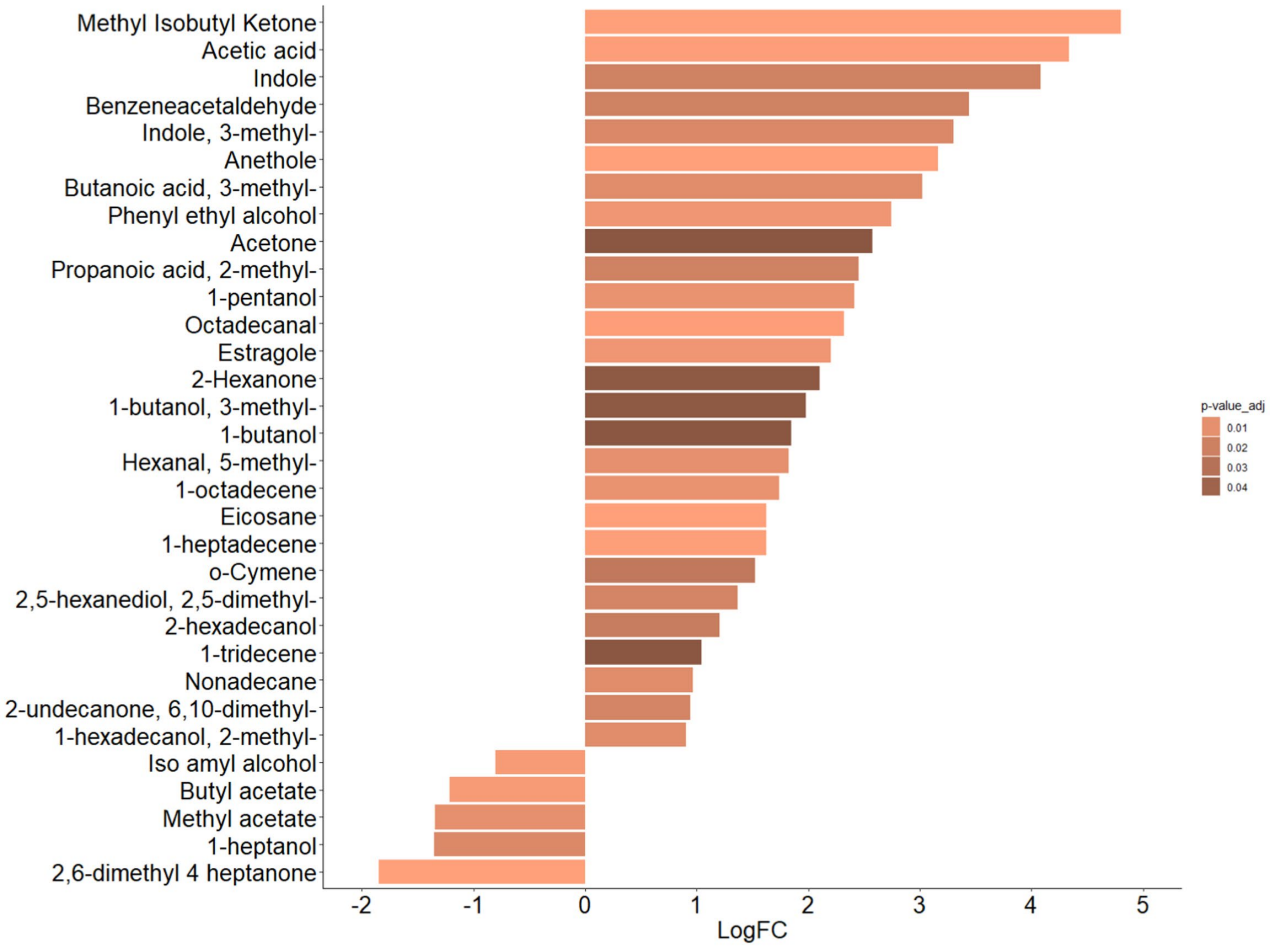
Bar plot of differential abundance of VOCs represented by Log_2_ of Fold Change (FC) for ASDs e CTRLs datasets. Bar plot represents the LogFC scores of differential abundance of VOCs (*p*-value adjusted ≤0.05, Mann–Whitney test). Color scale represents values of adjusted *p* value.

By exploiting univariate analysis, amongst the 32 statistically significant VOCs (FDR adjusted *p* value ≤0.05), represented below in the heat map (Supplementary Figure S4A), 27 VOCs were associated to ASDs and five VOCs to CTRLs, as quantitatively represented by the Fold Change (FC) bar plots (Figure 2).

In particular, the VOCs associated with ASDs were methyl-isobutyl ketone (*p* = 0.0001), acetic acid (*p* = 0.001), indole (*p* = 0.02), benzeneacetaldehyde (*p* = 0.02), 3-methyl indole (i.e., skatole) (*p* = 0.01), anethole (*p* = 0.0001), 3-methyl butanoic acid (*p* = 0.01), phenyl ethyl alcohol (*p* = 0.006), acetone (*p* = 0.05), 2-methyl propanoic acid (*p* = 0.02), 1-pentanol (*p* = 0.001), octadecanal (*p* = 0.001), estragole, (*p* = 0.006), 2-hexanone (*p* = 0.05), 3-methyl 1-butanol (*p* = 0.05), 1-butanol (*p* = 0.05), 5-methyl hexanal, (*p* = 0.01), 1-octadecene (*p* = 0.01), eicosane (*p* = 0.0003), 1-heptadecene (*p* = 0.001), o-cymene (*p* = 0.03), 2,5-dimethyl, 2,5-hexanediol (*p* = 0.02), 2-hexadecanol (*p* = 0.02), 1-tridecene (*p* = 0.05), nonadecane (*p* = 0.01), 6,10-dimethyl-2-undecanone (*p* = 0.02), and 2-methyl 1-hexadecanol (*p* = 0.01), and VOCs associated with CTRLs were iso amyl alcohol (*p* = 0.004), butyl acetate (*p* = 0.004), methyl acetate (*p* = 0.01), 1-heptanol (*p* = 0.02), and 2,6-dimethyl 4-heptanone (*p* = 0.0002).

Moreover, 24/27 VOCs (i.e., methyl isobutyl ketone, anethole, eicosane, 1-heptadecene, octadecanal, acetic acid, estragole, phenyl ethyl alcohol, 1-octadecene, 5-methyl hexanal, 1-pentanol, 3-methyl butanoic acid, 3-methyl indole, 2,5-dimethyl 2,5-hexanediol, 2-methyl propanoic acid, benzeneacetaldehyde, indole, 2-hexadecanol, o-cymene, 1-tridecene, acetone, and 1 butanol, 3-methyl 1 butanol, and 2-exanone), characterized by FC > 2, and 4/5 VOCs (i.e., methyl heptanone, butyl acetate, methyl acetate, and 1-heptanol), characterized by FC < −2, resulted in the most and least abundant, respectively, for the ASDs dataset (Supplementary Figure S4B).

The distribution of the 32 statistically significant VOCs (FDR adjusted *p* value ≤0.05), describing the ASDs vs. CTRLs comparison, and represented as hierarchical clustering heatmap, is reported in Supplementary Figure S4A. Indeed, a clear separation between patients and neurotypical subjects was revealed and reported as two major clusters, while two sub-clusters (namely A and B) were associated only to the ASD set.

Looking at anamnestic data, age (≤5 and > 5 years) and gender did not affect VOCs hierarchical clustering (Supplementary Figure S5). However, clinical data such as GI and severity symptoms, and disposition to be Pes, affected clustering topography. In particular, subjects without GI symptoms were mainly characterized by high expression of 1-tridecene, while subjects with high ASD severity symptoms were characterized by 2-butanamine and PEs by the most-abundant 3-methyl-1-butanol (Supplementary Figure S6).

Concerning the univariate analysis, anamnestic and clinical features were also considered to identify possible biomarkers associated with the patients’ stratification features. Hence, patients were grouped according to age (≤5 and > 5 years old) and gender. For age, nine metabolites (i.e., p-cresol, hexanoic acid, 2-octanone, octadecane, benzeneacetaldehyde, phenol, 2-nonanol, dimethyl disulphide, and butanoic acid) were statistically different between the two age subgroups and were all related to patients over 5 years of age (Supplementary Figure S7).

Regarding gender, statistically significant differences were reported only for 3-hexanone within the female subgroup, and for 1-heptadecene within the male subgroup (data not shown). By grouping patients for PEs vs. noPEs, 1-butanol-3-methyl was related to PE ASDs, while 2-nonanol, estragole, anethole, 2-hexanone, and citral were associated with noPE ASDs (*p* ≤ 0.05) (data not shown). By considering probiotics supplementation, four metabolites (i.e., hexanal, heptanal, 2-octanol, and 1-butanol-3-methyl) were statistically associated with ASD with probiotics supplementation and only one (i.e., octadecane) to ASD without probiotics supplementation (Supplementary Figure S8A). Moreover, 2-tridecanone was related to ASD patients treated with antibiotics, while *p*-cresol and benzaldehyde were statistically associated to the ASD subgroup without antibiotics administration (Supplementary Figure S8B).

**Figure 3:**
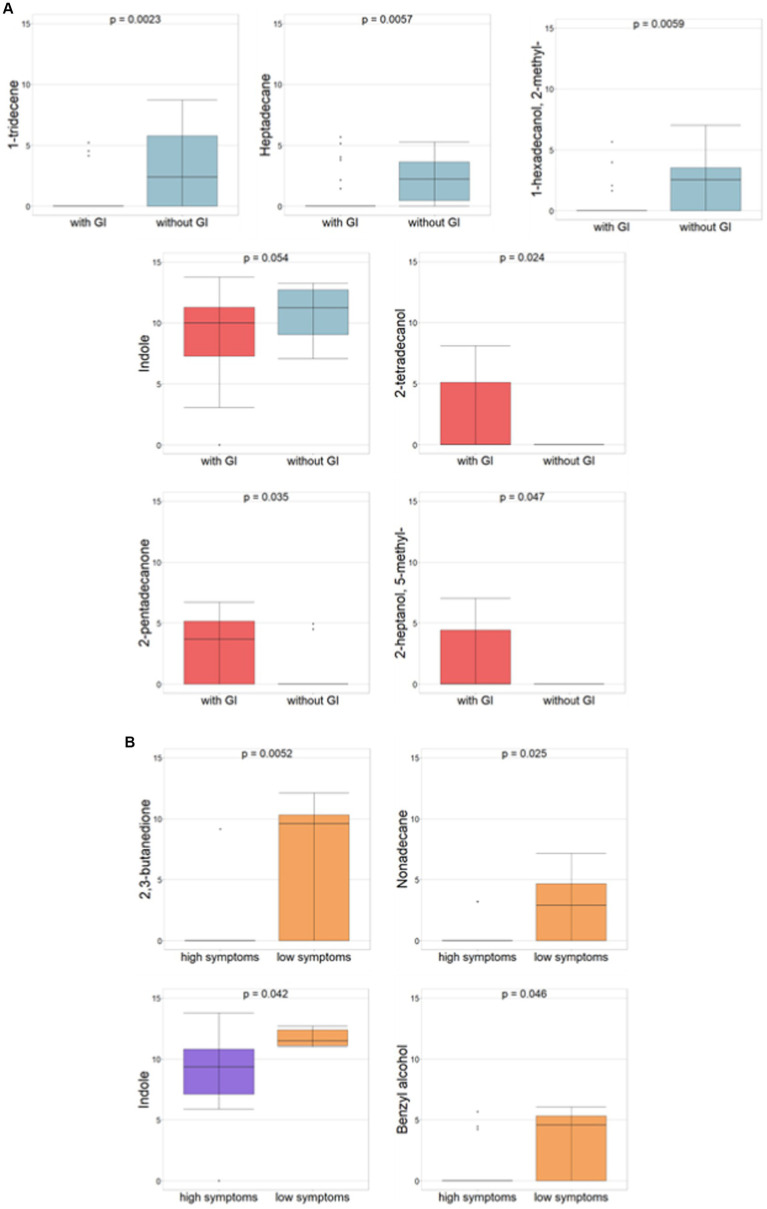
Differential abundances of VOCs (*p*-value ≤ 0.05, Mann–Whitney test) for ASDs subgrouped for GI and severity symptoms, respectively. **(A)** ASD with vs. ASD without GI symptoms. Red histograms refer to ASDs with GI; light blue histograms refer to ASD without GI. **(B)** ASDs with high autism vs. low severity symptoms. Violet histograms refer to high ASD symptoms; orange histograms refer to low ASD symptoms.

The comparison between ASD patients with and without GI symptoms also demonstrated statistically significant differences (*p* ≤0.05) for 2-tetradecanol, 2-pentadecanone, and 2-heptanol-5-methyl associated to ASDs with GI symptoms, however, 1-tridecene, heptadecane, 1-hexadecanol-2-methyl, and indole (*p* = 0.054) were related to ASDs without GI symptoms (Figure 3A).

Amongst neuropsychological features and according to autism severity (i.e., severe or mild, corresponding to high or low symptoms, respectively), 2,3-butanedione, nonadecane, indole, and benzyl alcohol all demonstrated statistical significance all of which are associated with ASD with low symptoms (Figure 3B).

Regarding behavioral problems, the evaluation of CBCL_INT and CBCL_EXT scale-based values, each including ASD patients with risk for behavioral problems, ASD patients with the presence of clinical symptoms, and ASD patients with no clinical symptoms, was exploited to consider the comparison between ASD patients with the presence of clinical symptoms vs. ASD patients with no clinical symptoms.

**Figure 4:**
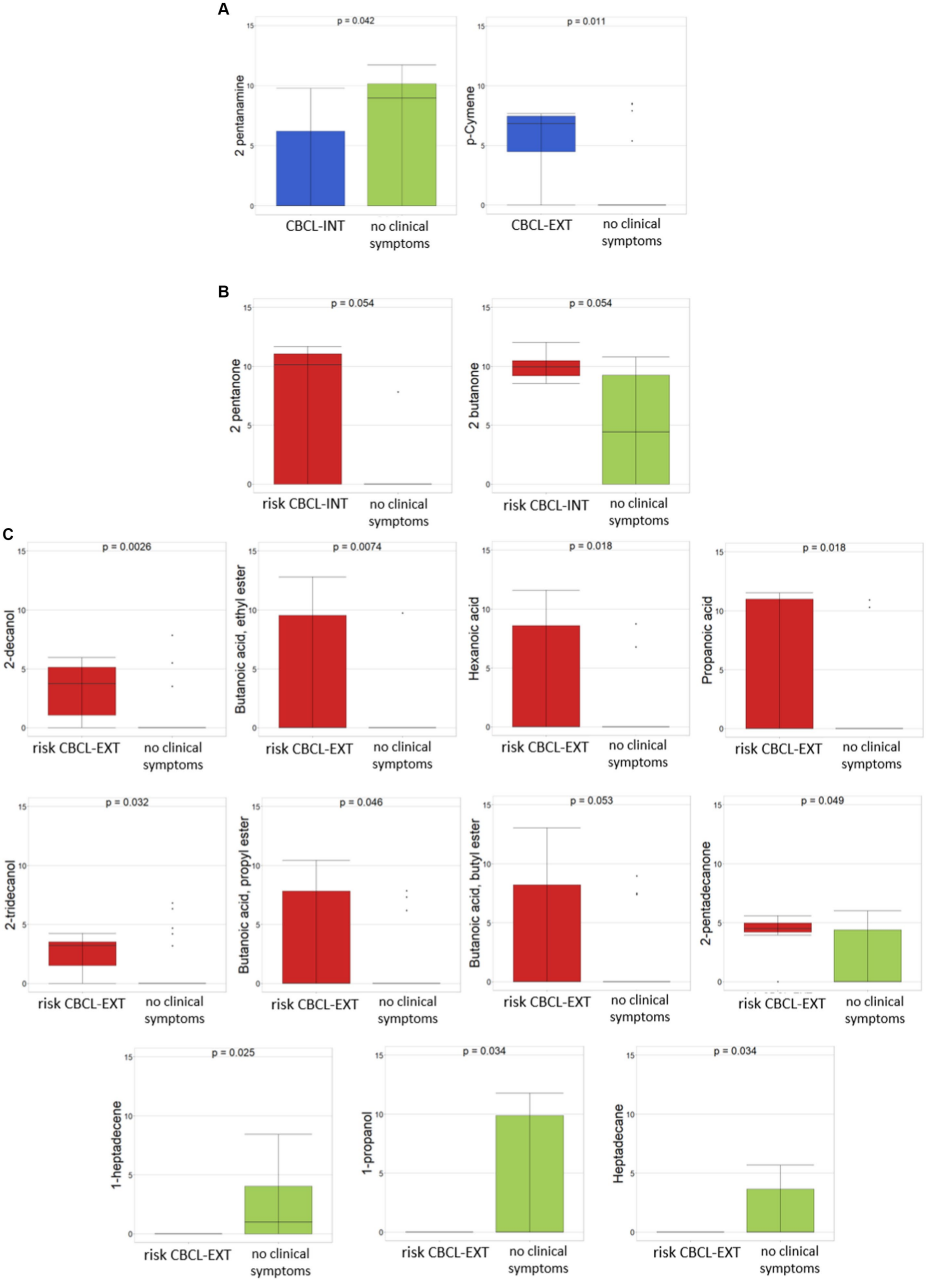
Differential abundances of VOCs between ASDs with clinical symptoms and ASD with risk of clinical symptoms, reported as CBCL-based score, vs ASD with no clinical symptoms. **(A)** CBCL-INT and CBCL-EXT, both related to ASDs with presence of clinical symptoms vs ASDs with no clinical symptoms. **(B)** CBCL-INT risk subgroup vs ASDs with no clinical symptoms. **(C)** CBCL-EXT risk subgroup vs ASDs with no clinical symptoms. Colors: **(A)** Blue, CBCL-INT and CBCL-EXT including ASDs with clinical symptoms; green, ASDs with no clinical symptoms; **(B)** and **(C)** red, CBCL-INT and CBCL-EXT including with ASDs with risk of clinical symptoms; green, ASDs with no clinical symptoms. *p* ≤ 0.05 based on Mann–Whitney test.

In particular, between CBCL_INT of ASDs with the presence of clinical symptoms (23/38) vs. ASDs with no clinical symptoms (8/38), the only statistically significant difference (*p* ≤0.05) was seen with 2-pentanamine, which was associated with ASDs with no clinical symptoms (Figure 4A). By considering the comparison between CBCL_EXT of ASDs with the presence of clinical symptoms (5/38) vs. ASDs with no clinical symptoms (26/38) (*p* ≤0.05), only p-cymene was statistically significant and related to CBCL_EXT of ASDs with the presence of clinical symptoms (Figure 4A). By considering the only CBCL_INT and CBCL_EXT risk subgroups, for the comparison between CBCL_INT risk group (7/38) vs ASD with no clinical symptoms (8/38), only 2-pentanone and 2-butanone were statistically associated (*p* ≤0.05) with the CBCL_INT risk group (Figure 4B). In addition, in the comparison between CBCL-EXT risk subgroup (7/38) vs. ASD with no clinical symptoms (26/38), 2-decanol, butanoic acid ethyl ester, hexanoic acid, propanoic acid, 2-tridecanol, butanoic acid propyl ester, butanoic acid butyl ester, and 2-pentadecanone were statistically significantly different, and were associated with the CBCL-EXT risk subgroup, while 1-heptadecene, 1-propanol, and heptadecane were related to ASDs with no clinical symptoms (Figure 4C).

For the other neuropsychological features, in particular pharmacological treatment and presence of epilepsy symptoms, a very low number of patients receiving pharmacological treatment and affected by epilepsy were present in the cohort, 6/41 and 3/41, respectively. Hence, comparisons were not robust (data not shown). Moreover, by considering comparisons based on cognitive level, reported as IQ/DQ with and without cognitive impairment or developmental delay, the statistically significant differences (*p* ≤ 0.05) were seen in 2,3 butanedione, 1-heptadecene, and 3-hexanone for the ASD group without cognitive impairment or developmental delay, and 2-tetradecanone for the ASD group with cognitive impairment or developmental delay (Supplementary Figure S9).

**Figure 5:**
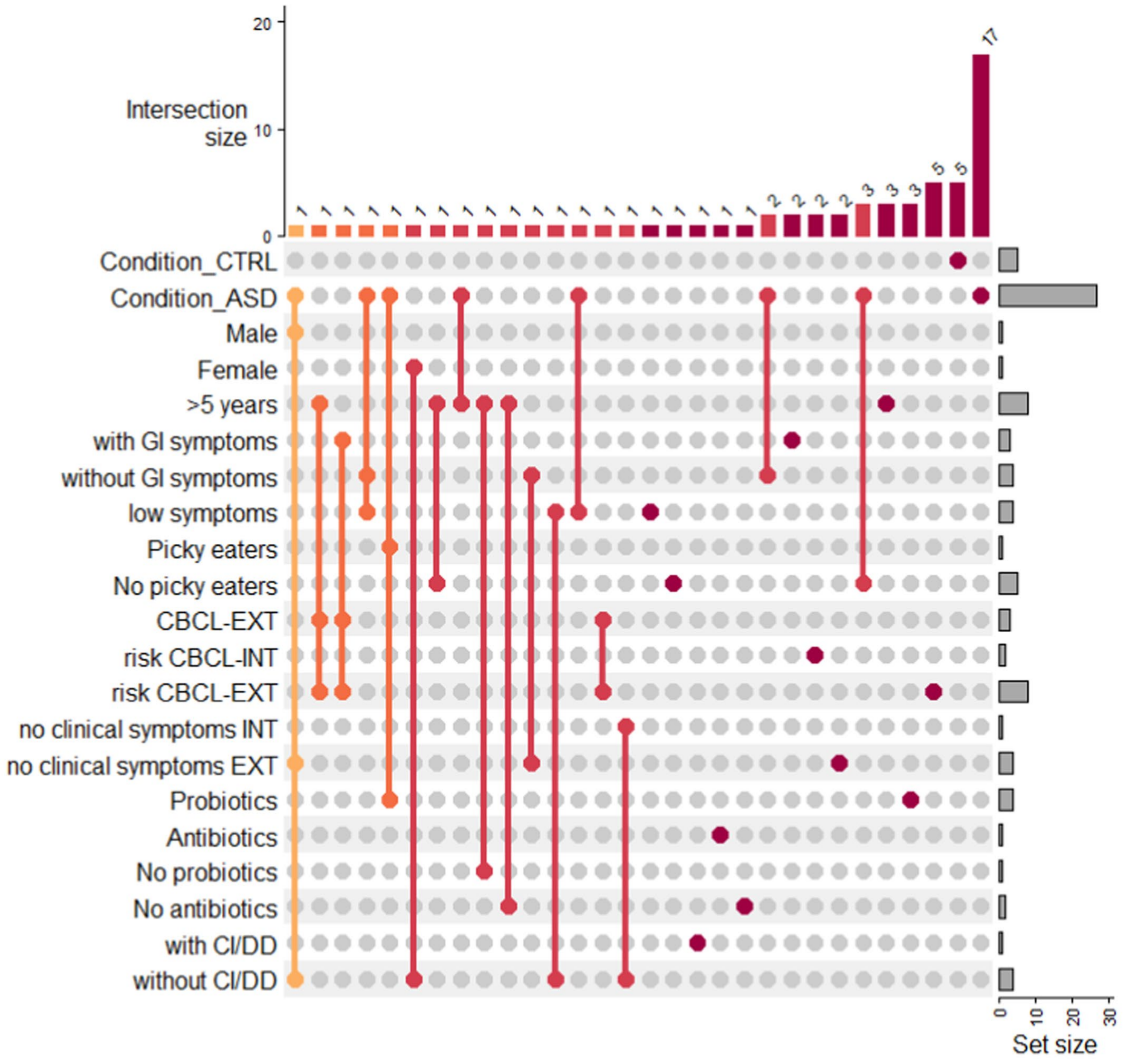
Upset plot of VOCs distribution in ASD subgroups and condition CTRL. Upset plot shows the distribution of statistically significant VOCs in the ASD subgroups and CTRL condition. The red/orange bar charts at the top represent the intersection size in the subgroups, while the grey bar charts represent the number of VOCs included in each ASD subgroup or in the CTRL condition. Legend: with CI/DD: presence of cognitive impairment/developmental delay; without CI/DD: absence of cognitive impairment/developmental delay.

The distribution of metabolites across neurotypical and ASD subgroups was visualized as UpSet plot (Figure 5; Supplementary Table S5). The UpSet plot shows the distribution of statistically significant VOCs in the ASD subgroups and CTRL condition. By considering a wide number of features, we have shown that the metabolites associated were only from 1 to 3. Conversely, for risk CBCL_EXT and CTRLs, the number of metabolites increase to 5 for both conditions (Supplementary Table S5). For patients with ASD, 17 metabolites were exclusively associated (Supplementary Table S5).

### 3.3 Metabolic pathways acting as discriminant players of GM in ASD and neurotypical subjects

**Figure 6:**
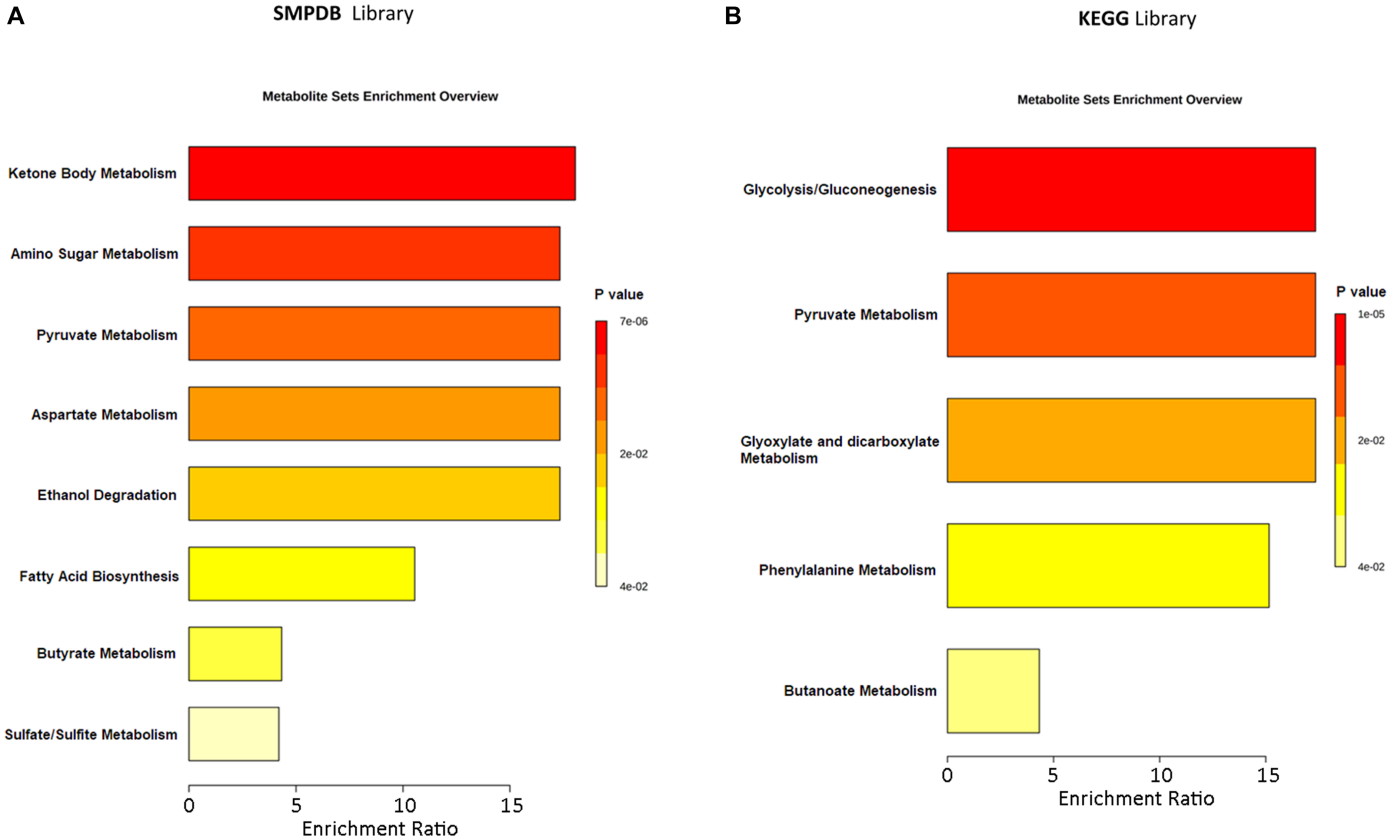
Metabolic Set Enrichment Analysis (MSEA) showing the most altered metabolic pathways in ASDs. **(A)** MSEA obtained by interrogation of SMPDB database. **(B)** MSEA obtained by interrogation of KEGG database. The length of each bar is dependent on the fold enrichment; the color intensity (from yellow to red) is proportional to statistical significance.

An MSEA was performed to identify the most statistically significant discriminant pathways between ASD patients and CTRLs. The discriminant pathways included ketone bodies, chemical molecules produced from fatty acids by the liver (ketogenesis), amino sugars, pyruvate, aspartate, glyoxylate/dicarboxylate, butyrate, sulfate/sulfite and phenylalanine metabolism, ethanol degradation, fatty acids biosynthesis, and glycolysis/gluconeogenesis (Figure 6; Supplementary Table S6). Among all these pathways, fatty acid metabolism (*p* = 0.036; impact = 0.025), generated by SMPDB, pyruvate (*p* ≤ 0.001; impact = 0.061), and phenyalanine metabolism (*p* ≤ 0.001; impact = 0.140), provided by the KEGG database, was most responsible for the separation between groups (Supplementary Figure S10; Supplementary Table S6).

### 3.4 Network correlation between microbial and metabolite signatures

**Figure 7:**
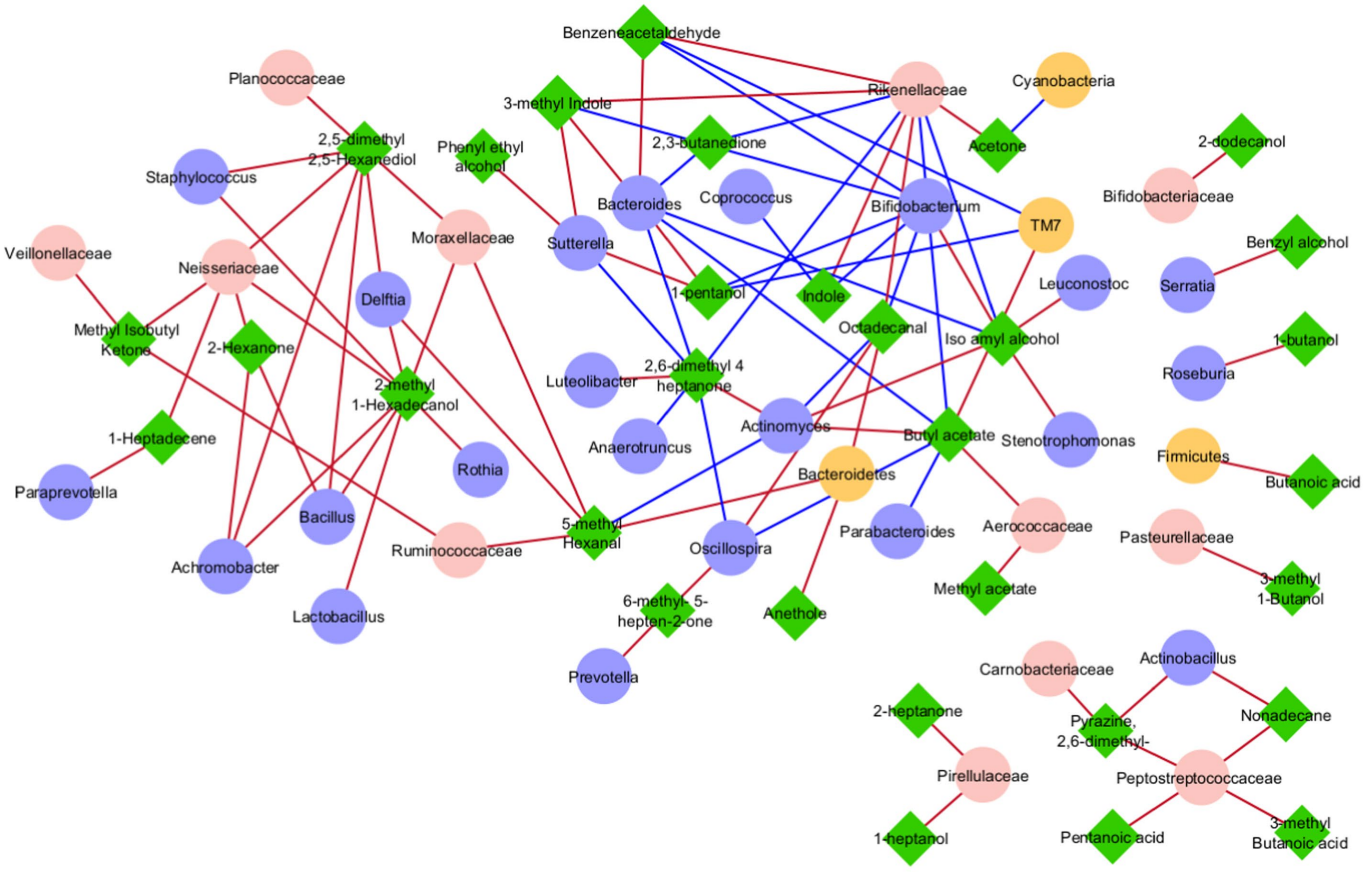
Correlation network analysis between OTUs and VOCs. In each network, nodes represent the OTUs (circles) and the VOCs (triangles), and an edge between two nodes occurs if they exhibit a statistically significant correlation (*p* ≤ 0.05). The color of the network edges indicates positive (red) and negative (blue) correlations.

The 589 OTUs and 110 VOCs from ASD and CTRL datasets were identified and processed to select the most important GM taxonomic lineages and VOC network correlations. The two datasets, including OTUs and VOCs filtered as statistically significant, were merged and a correlation matrix was obtained (Supplementary Table S7). Only statistically significant correlations were reported, and disconnected nodes were removed. At the end of the data processing, 38 OTUs and 31 VOCs were included in the network analysis (Figure 7). From this was formed four main groups: a major one, a small one, a triplet, and a quintet of couples (Figure 7). The small group was composed of OTUs and VOCs all with positive correlations, including Carnobacteriaceae, *Actinobacillus*, Pepetostreptococcaceae, pentanoic acid, 2.6-dimethyl-pyrazine, nonadecane, and 3-methyl-butanoic acid. The triplet showed two VOCs, 2-heptanone and 1-pentanol, and only one single OTU, Pirellulaceae, with positive correlations. Finally, the quintet was composed of OTUs-VOCs couples including Bifidobacteriaceae/2-dodecanol, *Serratia*/benzyl alcohol, *Roseburia*/1-butanol, Firmicutes/butanoic acid, and Pasteurellaceae/3-methyl 1-butanol with a positive correlation. Interestingly, in the major group, *Bifidobacterium* was negatively correlated with indole and skatole (Supplementary Table S7; Figure 7). By considering only the ASD patients group, for which 140 statistically significant correlations amongst OTUs and VOCs were obtained, 54 correlations were negative and 86 positive (Supplementary Table S7).

### 3.5 Integrated network WGCNA analysis amongst OTUs, VOCs, and clinical data

**Figure 8:**
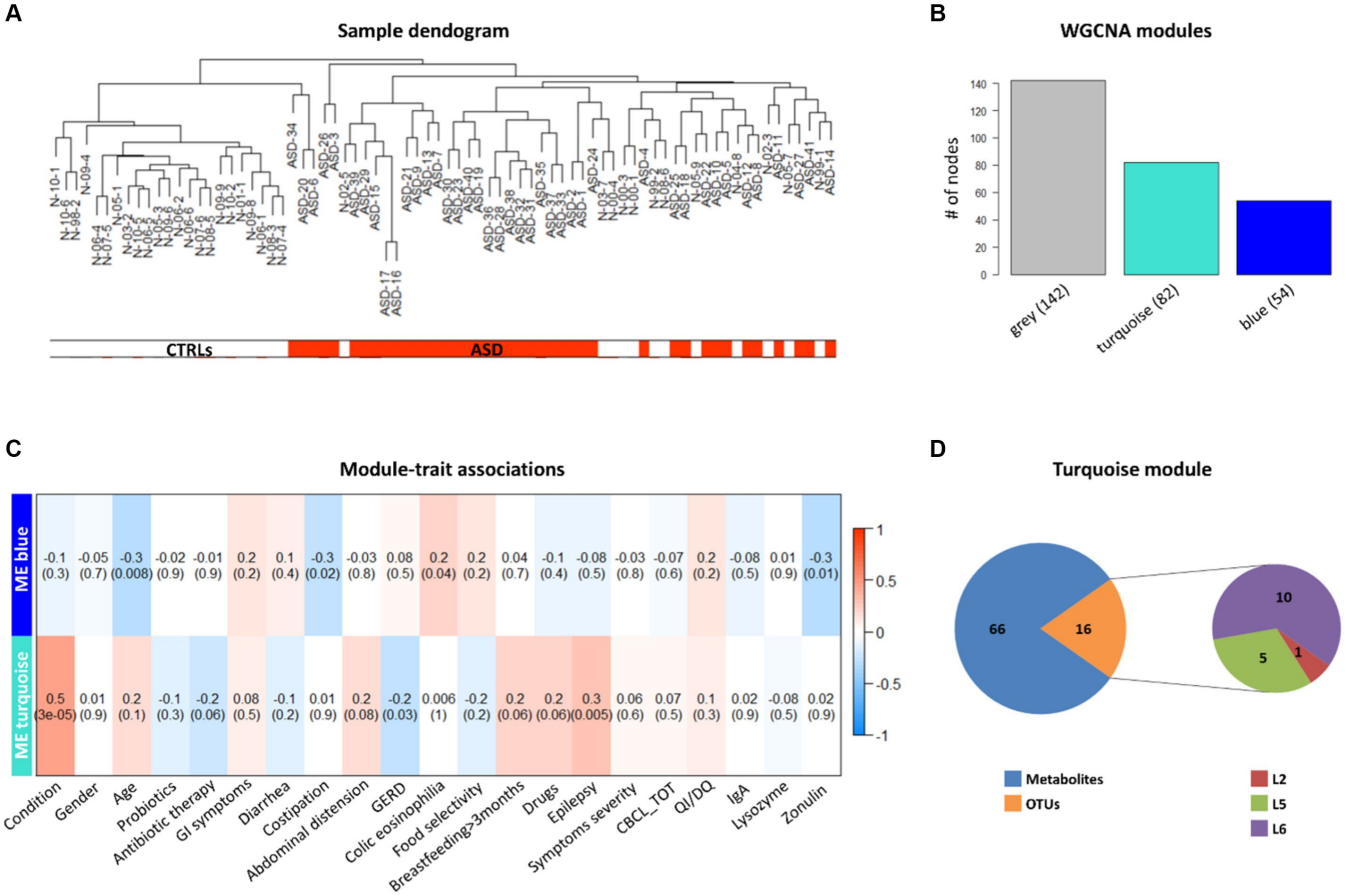
Weighted correlation network analysis (WGCNA) of OTUs, VOCs, and clinical data of ASD patients and CTRLs. **(A)** Clustering dendrogram of ASD and CTRL samples. The horizontal bars represent how the ASD and CTRL condition relates to the sample dendrogram: white annotation refers to CTRLs (low distance values) and red to ASD patients (low distance values). **(B)** WGCNA modules. The bars represent the size (i.e., number of nodes) of each WGCNA-detected module, colored with different module labels (i.e., grey, turquoise, blue). **(C)** associations between clinical trait or feature and ME, represented by correlation heatmap. In the heat map, each row corresponds to a given ME and each column to a trait or feature of interest. Each cell contains the correlation and (within the round brackets) the associated *p* value between them. The heatmap’s color-coded by correlation according to the color legend. **(D)** WGCNA turquoise module composition. Pie charts represent the numbers of 16 OTUs (one phylum, five families and 10 genera) and 66 VOCs falling in the module.

To understand the potential correlation between the GM volatilome, ecology, and patients’ clinical traits, WGCNA analysis was applied to identify VOC and OTU biomarkers. Firstly, sample clustering was conducted to detect outliers. All samples, namely 41 ASDs and 35 CTRLs, passed the cut-off threshold and two main clusters were identified, the first one mainly corresponding to ASDs and the second one mainly including CTRLs (Figure 8A). The weighted correlation network identified three network modules labeled by color (Figure 8B) with the grey module grouping all nodes with outlying profiles and, henceforth, not considered. Tests of association between each clinical trait or feature (i.e., disease conditions, gender, age, probiotics, antibiotics, GI symptoms, nutritional habits, birth modality, feeding, epilepsy, autism severity, neurological screening CBCL scales, IQ/DQ, IgA, zonulin, and lysozyme) and each ME were performed and represented by heatmap (Figure 8C). For each node in each module, the values of MM and BS, with respect to each significant trait or condition, were calculated (Supplementary Table S8). Interestingly, the turquoise module appeared to show the highest statistically significant (positive) correlations with ASD vs. neurotypical condition and epilepsy (Figure 8C). These correlations predominantly included VOCs for both ASDs and CTRLs (Supplementary Table S8). The largest number of significant biomarkers (i.e., 23) of ASDs were, among others, methyl isobutyl ketone, acetic acid, acetone, indole, iso amyl alcohol, skatole, phenol, and butanoic acid (Supplementary Table S8). The few statistically significant OTUs involved in the correlation (*p* ≤ 0.05) (Figure 8D), were mainly abundant in CTRLs rather than ASDs. Indeed, the unique OTUs involved in the turquoise module, and assigned by BS, were Erysipelotrichaceae, Lachnospiraceae, and the genus *Coprococcus*, and were associated to CTRLs (Supplementary Table S8), confirming the previous work on ecological patterns (Vernocchi et al., 2022).

### 3.6 Gut microbiota VOCs: role as predictors

**Figure 9:**
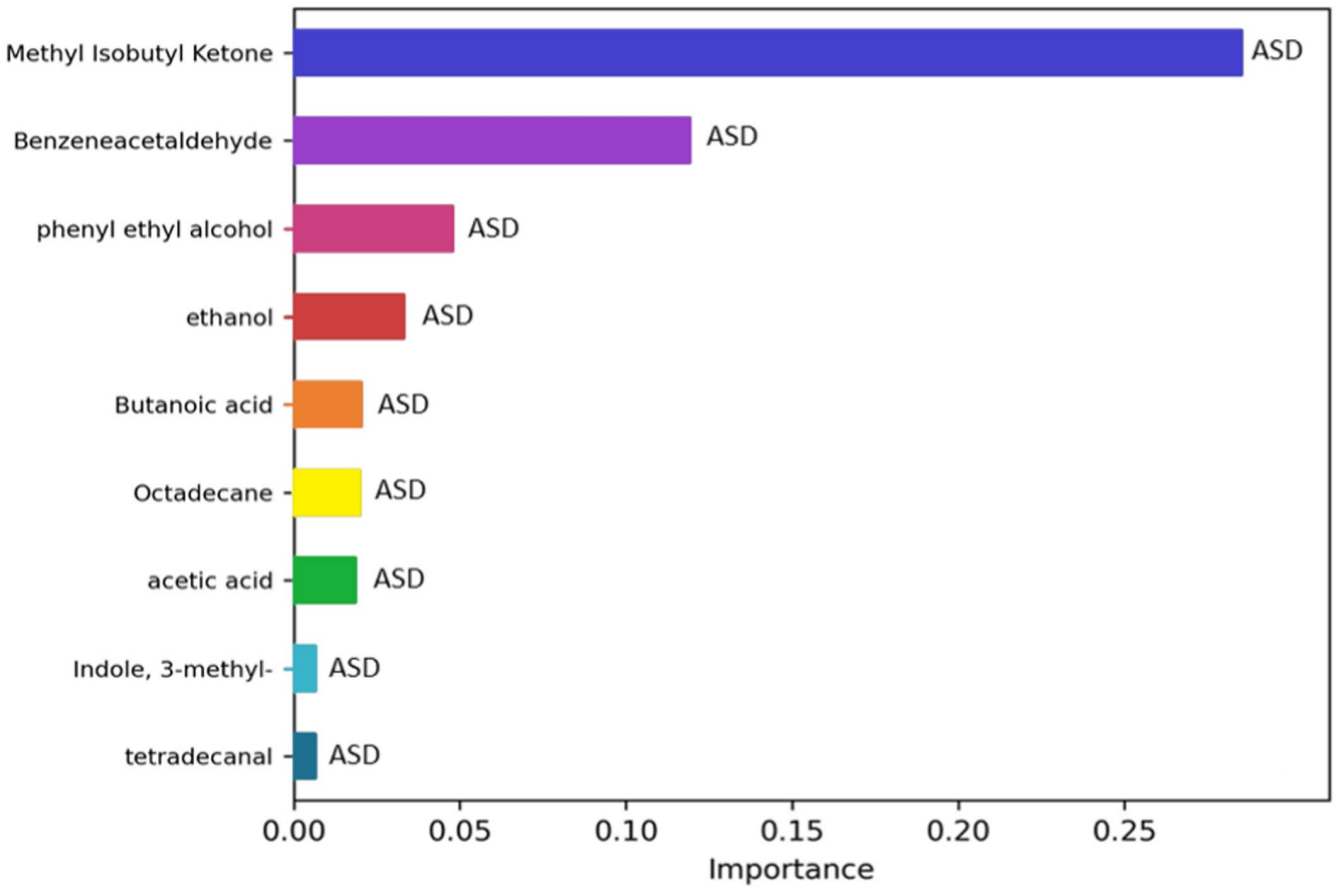
Significant metabolites selected by the classification model HistGradientBoostingClassifier. The bar charts represent the importance scores of each VOC in the ASD prediction models compared to CTRLs.

To investigate if the GM volatilome of ASD patients could be predictive of neurodivergence, classification analyses based on ML were exploited. The most important features of the GM volatilome, able to classify 80–89% of patients compared to CTRLs, were identified based on the top performing five models: GaussianNB, ExtraTreesClassifier, AdaBoostClassifier, MLPclassifier, and HistGradientBoostingClassifier. The latter was the best performing model, characterized by an accuracy of 89%. The VOCs associated with ASDs were methyl isobutyl ketone, benzeneacetaldehyde, phenyl ethyl alcohol, ethanol, butanoic acid, octadecane, acetic acid, skatole, and tetradecanal (Figure 9).

Receiver operating characteristic (ROC) analysis and logistic regression models were used to observe the association of VOCs with ASD severity, particularly by considering the metabolites with statistically significant changes between ASD patients and CTRLs. Amongst the 32 VOCs filtered by Wilcoxon test and shown in the heat map (Supplementary Figure S4A), of which 27/32 were most abundant in ASDs and 5/32 in CTRLs (Supplementary Figure S4B), 12 VOCs were characterized as statistically significant (*p* ≤ 0.05), and all of them were most abundant in ASD patients. Moreover, based on the logistic regression model, 6/12 VOCs were associated with ASD. Positive odds for each VOC were 1.33 (methyl isobutyl ketone), 1.40 (phenyl ethyl alcohol),1.18 (indole), 1.22 (3-methyl indole), 1.20 (acetic acid), and 1.31 (benzeneacetaldehyde), suggesting that these six VOCs were accurate as predictors in discriminating the ASD group (Supplementary Figure S11).

## 4 Discussion

The bioavailability and utilization of substrate-derived metabolites in the gut are affected by microbial communities that have an impact on the host’s metabolic function (Neis et al., 2015). Specifically, saccharolytic and proteolytic fermentation exerted by gut microbes contributes to the production of branched chain fatty acids (BCFA) and SCFAs, alcohols, ketones, ammonia, poly- and mono-amines, indoles, phenols, sulfide, and others metabolites (Jardon et al., 2022).

This research aims to deepen our understanding of how microbiota-derived metabolites interact with the host, and eventually compromise or maintain intestinal homeostasis, potentially affecting neurodevelopment and affecting the onset and progression of neurological disorders such as ASD (Peralta-Marzal et al., 2021). In this context, untargeted metabolomics may contribute to biomarker discovery, highlighting how the GM metabolome may indeed influence neurological disorders (Schwarcz and Stone, 2017; Su et al., 2022; Ye et al., 2022).

The human volatilome included a wide mixture of volatile releases produced by the human body and its microbiomes (Elmassry and Piechulla, 2020). Particularly, microbes make VOCs as products of main and ancillary metabolic pathways. Crosswise microbial kingdoms, the metabolism of lipids, sugars, amino acids, sulfur- and nitrogen-compounds, and aromatic compounds, and their metabolism contribute to the production of thousands of VOCs (Ryu et al., 2020; Weisskopf et al., 2021).

Hence, several studies have utilized untargeted screening of volatile metabolites to discriminate patient and control groups. However, common microbial metabolites have also been detected through different disease-associated groups, and several studies have evidently demonstrated translation of *in vitro* microbial volatilomics through clinical samples (Elmassry and Piechulla, 2020).

The current study regards the GM volatilome profile of 41 children with ASD, fully characterized from a neuropsychological point of view, in terms of symptom severity and comorbidities, such as GI functional symptoms (Chaidez et al., 2014; Madra et al., 2021), and compared to 35 neurotypical subjects, referred to as CTRLs.

Specifically, in our study, the GM core volatilome for all ASD patients was characterized by over-production of 1-pentanol, 1-butanol, phenyl ethyl alcohol; benzeneacetaldehyde, octadecanal, tetradecanal; methyl isobutyl ketone, 2-hexanone, acetone; acetic, propanoic, 3-methyl-butanoic and 2-methyl-propanoic acids; indole and skatole; and o-cymene. The GM volatilome of neurotypical subjects was mainly characterized by butyl acetate and methyl acetate esters, consistent with previous studies (De Angelis et al., 2013, 2015). SCFAs, particularly butanoic and acetic acid, can be produced by *Faecalibacterium, Roseburia, Sutterella*, and *Prevotella* (Vernocchi et al., 2022), all of which are starch-degrading and carbohydrate-fermenting bacteria, and which may actually preserve GM ecology (Tamanai-Shacoori et al., 2017; Gálvez et al., 2020). SCFAs do not act as a neuroactive substances class, but they play a pivotal role in preserving neurotransmitters (Coretti et al., 2018), inducing modifications in gene expression related to neurotransmitter systems, neuronal cell adhesion molecules, FA and lipid metabolism, inflammation, and mitochondrial function oxidative stress, all having possible relevance in autism (Nankova et al., 2014).

Indeed, in animal models it has been detected that low doses of butyrate produce positive effects on the brain and behavior, while high doses induce a stress-like response (Gagliano et al., 2014; Kratsman et al., 2016; Coretti et al., 2018). In our patient cohort, butanoic acid was detected by WGCNA and ML as a biomarker associated with ASD, while the univariate analysis identified it as a biomarker associated with ASD patients specifically over 5 years of age, consistent with the hypothesis of pathogenetic effects triggered by butanoic acid in autism (Gagliano et al., 2014). Moreover, a bi-directional perturbation of the dopaminergic pathways by both propanoic and butanoic acids has been described, suggesting that a similar dysregulation of brain catecholaminergic system occurs, as a response, in the presence of excessive concentrations of SCFA (Nankova et al., 2014).

In our patient cohort, propanoic acid was associated with the CBCL_EXT scale for ASDs with risk of symptoms, compared to ASDs without clinical symptoms, hence suggesting a role for SCFAs in the manifestation of behavioral problems. Since propanoic acid passes the gut-blood and blood–brain barriers, it could travel from the intestine to the CNS (Mepham et al., 2019). Moreover, propanoic acid has been shown to induce hyperactivity, monotonous behaviors, impaired social behavior, increased repetitive locomotor activity, caudate spiking, and an innate neuroinflammatory response in ASD patients (Zwaigenbaum et al., 2005; MacFabe, 2012, 2015; Liu et al., 2019; Mirzaei et al., 2021). High concentrations of SCFA also downregulate the expression of genes involved in the biosynthesis and degradation of dopamine, norepinephrine, and serotonin (Ebert et al., 1997). Particularly, butanoic acid operates as a powerful inhibitor of histone deacetylase in the regulations of the neurotransmitters norepinephrine, dopamine, and epinephrine, and also modulates the inflammatory and oxidative conditions of intestinal mucosa (MacFabe, 2015). It is possible to assume that epigenetic regulation of gene expression via GM-derived SCFAs could result in “loss” or “increase” functions and in the modification of pathways/networks, such as the genetic modifications related to ASD (Nankova et al., 2014).

Furthermore, tryptophan derivatives such as indole and skatole were significantly increased (*p* ≤ 0.05) in children with ASD, particularly skatole, and thus were also identified as biomarkers by the ML predictive model. Only a small proportion of ingested tryptophan can be metabolized by human host cells (kynurenine pathway and serotonin pathway) and the remaining part by symbiotic intestinal bacterial (indole and its derivatives pathway) (Gostner et al., 2020; Konopelski and Mogilnicka, 2022). Recent research outcomes are providing evidence that GM-derived metabolites from tryptophan share the biological properties of their precursors (Konopelski and Mogilnicka, 2022). It has been hypothesized that indole is an inter-kingdom signal in gut epithelial cells, reinforcing the host cell-barrier assets (Bansal et al., 2010). It is derived from tryptophan, produced by several microbes (i.e., *Bacteroides*, *Clostridium*, *Desulfovibrio*) colonizing the human GI tract (Persico and Napolioni, 2013), and is a critical precursor of physiologically important molecules, such as serotonin and melatonin (De Angelis et al., 2013).

Consistent with this evidence, it was found that indole and skatole concentrations were increased in children with ASD (De Angelis et al., 2013). However, indole was more represented in ASD subgroups characterized by low severity symptoms and without GI symptoms, hence suggesting a GI protective role and a quenching effect on the neuropsychological conditions, that could act as a kind of protective agent. Particularly, indole and its derivatives seem to increase the integrity of the epithelial barrier and function of tight junctions (Bansal, 2012; Shimada et al., 2013) and to reduce colitis related to *Citrobacter rodentium* and *Candida albicans* infection (Bommarius et al., 2013). Moreover, GM indole derivatives may influence the level of serotonin precursors that affect the amount of serotonin in the brain (Agus et al., 2018). However, high levels of oxindole and isatin, after the epithelial and hepatic enzymatic digestion of indole in blood and urine, respectively, have been detected in hepatic encephalopathy and Parkinson’s disease (Hamaue et al., 2000).

Therefore, it has been hypothesized that an excessive production of indole by GM, which may be due to a gut dysbiosis or to specific GM composition signatures, could emerge in increased brain exposure to oxindole and isatin, affecting behavior and thus defining another pathway whereby indole could act as a signal to the brain and cause behavior alterations (Jaglin et al., 2018). In addition, it has been suggested that subjects whose GM is highly inclined to produce indole could be more likely to develop anxiety or depressive disorders (Jaglin et al., 2018). Moreover, the principal communication pathway between gut bacteria and the brain is recognized to be the vagus nerve (Bravo et al., 2011; Kelly et al., 2015), and the metabolites originating from the GM, such as butyrate, seem to stimulate the vagus nerve (Stilling et al., 2016). Thus, it is conceivable that this route represents a possible signaling pathway for indoles to the brain (Jaglin et al., 2018).

Finally, other potential metabolic biomarkers in ASD were represented by alcohols (ethanol, phenyl ethyl alcohol, 1-pentanol, 3-methyl-1-butanol, etc.). Alcohols promote dysbiosis and gut permeability and may alter the mucosal tight junctions and the immune activity in the GI tract, leading to a modification of gut barrier integrity, which allows microbial products such as indoles and FA to cross into the circulatory system (Samuelson et al., 2019). Alcohols were also reported to alter the GM composition, stimulating the growth of Gram-negative facultative anaerobes producing exotoxins (i.e., lipopolysaccharides, LPS) (Duan et al., 2019). These products, via toll-like receptors (TLRs), may enhance inflammatory activity, fibrosis, and cell death, probably being mediators of alcohol-related organ damage (Carpita et al., 2022). In addition, aldehydes such as benzenacetaldehyde were thought to be involved in the phenylalanine pathway (Piras et al., 2022)^1^ and have been highlighted by the ML approach as potential ASD biomarkers.

Aldehydes and ketones produced by bacteria have been reported to have both beneficial and harmful effects (Salaspuro, 1997; Mu et al., 2018). These metabolites can be generated endogenously as a result of oxidative stress via lipid peroxidation, as they are dietary components, and have been related to diabetes, cancer, and neurodegenerative disorders (Xie et al., 2016).

Specifically, for methyl isobutyl ketone, the highest ketone associated with ASD, we assume that its presence probably derived from the gut microbial imbalance of patients. This metabolite has also been described for nonalcoholic fatty liver disease (NAFLD)/nonalcoholic steatohepatitis (NASH) (Del Chierico et al., 2017) and for Juvenile Idiopathic Arthritis (JIA) (Vernocchi et al., 2020).

Regarding the enrichment pathway analysis, several pathways overexpressed in ASD patients, such as ketone body metabolism, butyrate, sulfate/sulfite and phenylalanine metabolism, and FA biosynthesis, actually characterized the patient group. Ketone bodies are produced primarily in the liver from β-oxidation and are transported to extrahepatic tissues for final oxidation. The principal ketone bodies are represented by acetoacetic acid, β-hydroxybutanoic acid, and acetone, which play key roles as signaling molecules, affect protein post-translational modification (PTM), and act as mediators of inflammation and oxidative stress (Puchalska and Crawford, 2017).

Hence, ketone body metabolism can be involved in the inflammation and in the oxidative stress that may be one of the mechanistic origins of neuroprogressive disorders (i.e., depressive and bipolar disorder or schizophrenia) (Guo et al., 2022), hence reflecting advanced neuroanatomical and cognitive degeneration, caused by several factors such as inflammation, peripheral, brain, and oxidative stress, and mitochondrial dysfunction correlated with tryptophan metabolism disorders (Berk et al., 2011).

Furthermore, the high expression of phenylalanine metabolism suggests some association between ASD and unusual gut bacterial metabolism of phenylalanine, as inferred by Vernocchi et al. (2022), in which the overexpression of quinate/shikimate dehydrogenase in children with ASD, involved in the production of aromatic AA, such as L-phenylalanine and L-tryptophan by Proteobacteria, were inferred. Additionally, an increase of phenylacetylglycine has been detected in the urine of children with ASD children, indicating a consistent increase of gut permeability that could be linked to phenylalanine accumulation, stimulated by bacterial aromatic amino acid biosynthesis via the shikimate pathway (Mir et al., 2015).

Phenol compounds were increased in the stool of children with Pervasive Developmental Disorder Not Otherwise Specified (PDD-NOS) and, especially, ASD (De Angelis et al., 2013). Consistently with this evidence, our data showed phenol and *p*-cresol were higher in ASD patients (*p* ≥ 0.05) compared to CTRLs. These are metabolites deriving from amino acids (AAs) such as tyrosine and phenylalanine (Vanholder et al., 1999). Particularly, *p*-cresol is hypothesized to exacerbate ASD severity and gut disorders, in the presence of intestinal infection, antibiotic consumption, and atypical intestinal permeability considered as potential *p*-cresol excess sources in ASD (Persico and Napolioni, 2013).

To date, the pathophysiology of ASD is still unclear and there are no specific treatments designed for it. Taken together with the previously published ecological study on children with ASD (Vernocchi et al., 2022), it seems that some microbial taxa (i.e., *Bacteroidetes*, *Proteobacteria, Roseburia, Sutterella, Prevotella, Faecalibactrium*, etc.) and some VOC (i.e., indoles, skatole, phenol, SCFAs, benzeneacetaldehyde, methyl isobutyl ketone, and ethanol) levels might be potential biomarkers to discriminate ASDs from neurotypical children. Moreover, the results of our cross-cohort analysis suggest that influencing factors, particularly host age, GI hallmarks, and autism severity, should always be taken into consideration to establish GM-related biomarkers of disease.

Finally, it is possible to assume that autism pathogenesis is related to both gut microbial ecosystem ecology and to signaling molecules of bacterial origin (Finegold et al., 2002).

Overall, our findings highlight the presence of altered microbial metabolites, as well as potential neuroactive effects, by which gut-derived SCFAs, indole derivatives, and other molecules could impact disease onset and progression. Indeed, indole, skatole, and butanoic and propanoic acids need to be re-assessed in this disorder as potential ASD phenotypes, related to GM metabolic activity, and, hence, to molecules that may play a potential role as postbiotic treatments.

All GM-related computational approaches, based on prediction capabilities of ML-based models, may play a pivotal role for the development of new strategies for ASD diagnostic assistance with a smart approach, such as clinical decision support systems (CDSSs) (Ristori et al., 2020). The progressive identification of new metabolites acting as biomarker candidates, combined with patient genetic and clinical data and environmental factors, including GM, would bring us towards advanced CDSSs, assisted by ML models for advanced ASD-personalized medicine, based on omics data integrated into electronic health/medical records. Furthermore, new ASD screening strategies based on GM-related predictors might be used to improve ASD riskiness assessment. Enhanced understanding of GM-related metabolites may bring new insights into ASD onset and progression, as well as leading to the discovery of new risk predictors.

## Data availability statement

The datasets presented in this study can be found in online repositories. The names of the repository/repositories and accession number(s) can be found below: https://www.ncbi.nlm.nih.gov/bioproject, PRJNA754695.

## Ethics statement

The studies involving humans were approved by Bambino Gesù Ethical Committee. The studies were conducted in accordance with the local legislation and institutional requirements. Written informed consent for participation in this study was provided by the participants’ legal guardians/next of kin.

## Author contributions

PV: Conceptualization, Data curation, Formal analysis, Investigation, Methodology, Supervision, Writing – original draft, Writing – review & editing. CM: Formal analysis, Writing – review & editing. SGu: Investigation, Writing – review & editing. FDC: Data curation, Writing – review & editing. VG: Formal analysis, Methodology, Writing – review & editing. SGa: Methodology, Writing – review & editing. FC: Formal analysis, Validation, Writing – review & editing. PP: Validation, Writing – review & editing. GI: Data curation, Investigation, Writing – review & editing. AG: Supervision, Writing – review & editing. SV: Funding acquisition, Investigation, Project administration, Writing – review & editing. LP: Conceptualization, Funding acquisition, Investigation, Project administration, Supervision, Validation, Writing – original draft, Writing – review & editing.

## Footnotes

^1^https://www.genome.jp/pathway/phs00360
