# Supplementary material for: Gut microbiota functional profiling in autism spectrum disorders: bacterial VOCs and related metabolic pathways acting as disease biomarkers and predictors
Source: Front Microbiol. 2023 Dec 18;14:1287350. doi: 10.3389/fmicb.2023.1287350 (PMC10773764; doi:10.3389/fmicb.2023.1287350)
Supplement: Supplementary file 9 [file Data_Sheet_1.docx]

**
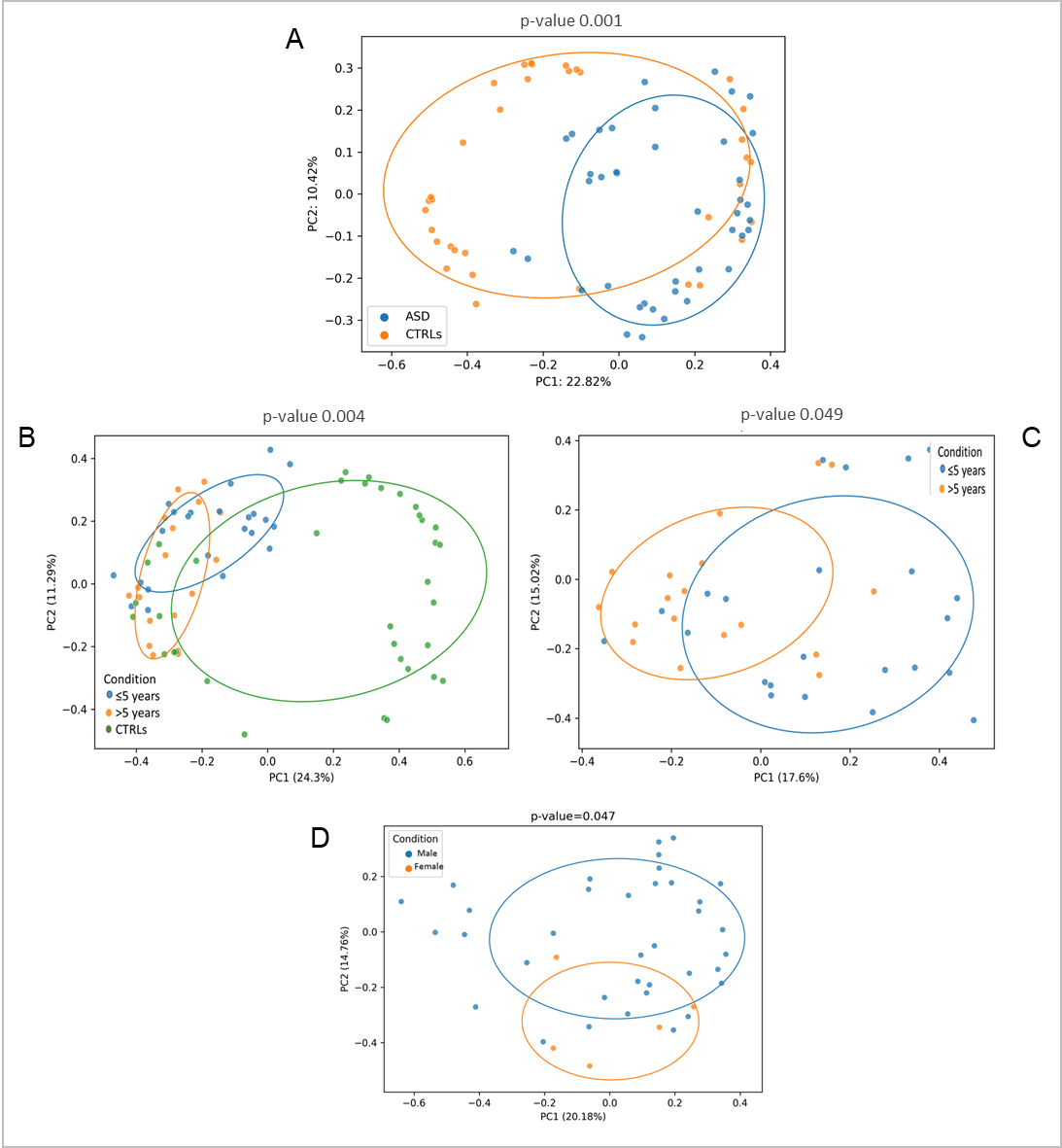
**

**Supplementary Figure 1.** **Beta-diversity of volatilome data. Principal Coordinate Analysis (PCoA) plots show Bray Curtis dissimilarity**. **Panel A,** ASDs *vs.* CTRLs; **Panel B,** ASDs subgrouped for age (≤5 or >5 years) compared to CTRLs; **Panel C**, ASDs subgrouped for age (≤5 or >5 years). **Panel D**, ASDs subgrouped according to gender.


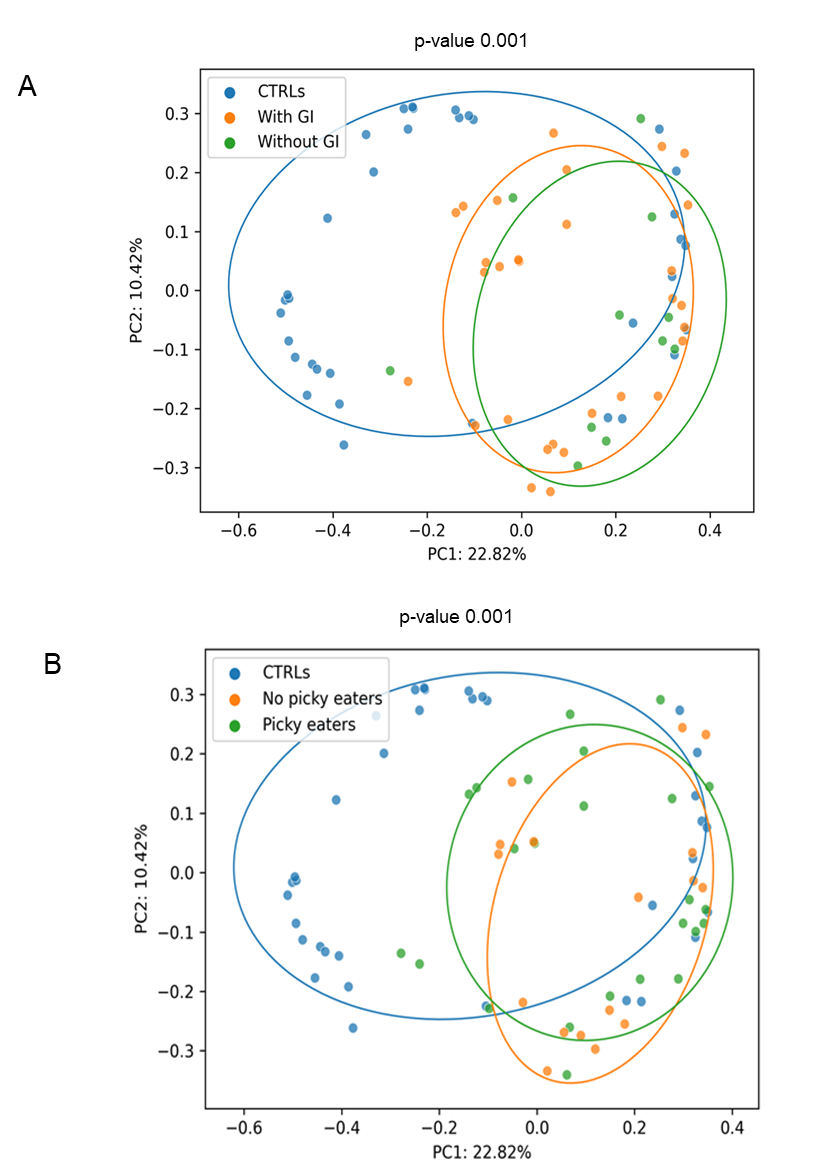


**Supplementary Figure 2.** **Beta-diversity of volatilome data. Principal Coordinate Analysis (PCoA) plots show Bray Curtis dissimilarity**. **Panel A,** ASDs subgrouped for GI symptoms (with GI or without GI) compared to CTRLs; **Panel B,** ASDs subgrouped for disposition to be Picky eaters (PEs and no PEs) compared to CTRLs.


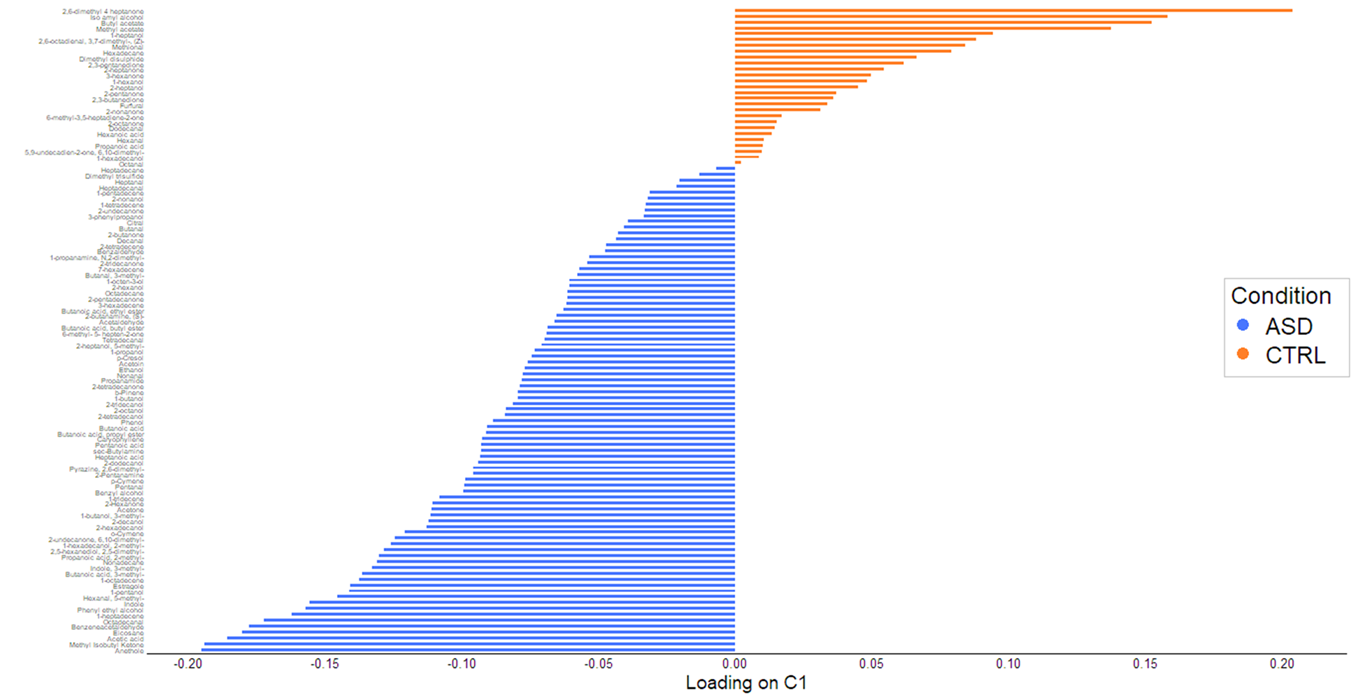


**Supplementary Figure 3**. Loadings PLS-DA: most important metabolites contributing to the model between ASD and CTRLs. Increased metabolites are marked in red, decreased ones in blue

.


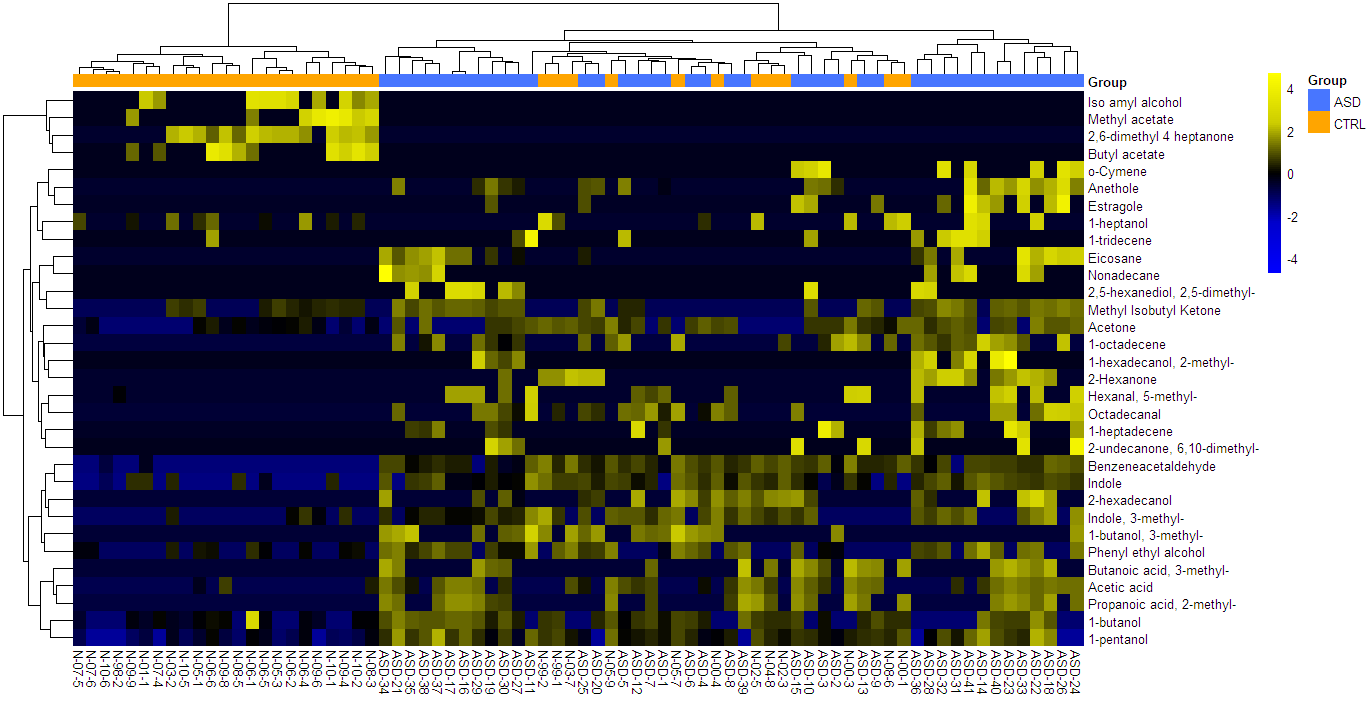


1

2

A

B

**Supplementary Figure 4A**. **Heatmap of statistically significant VOCs** between **ASD**s and **CTRLs** constructed using Euclidean distance and Ward's clustering method. The color scale characterizes the Z- score values for each variable: yellow, high level; blue, low level. The column bar colors represent the subject condition category: blue, ASD; orange, CTRLs. The p value ≤ 0.05 is corrected with FDR method.

**
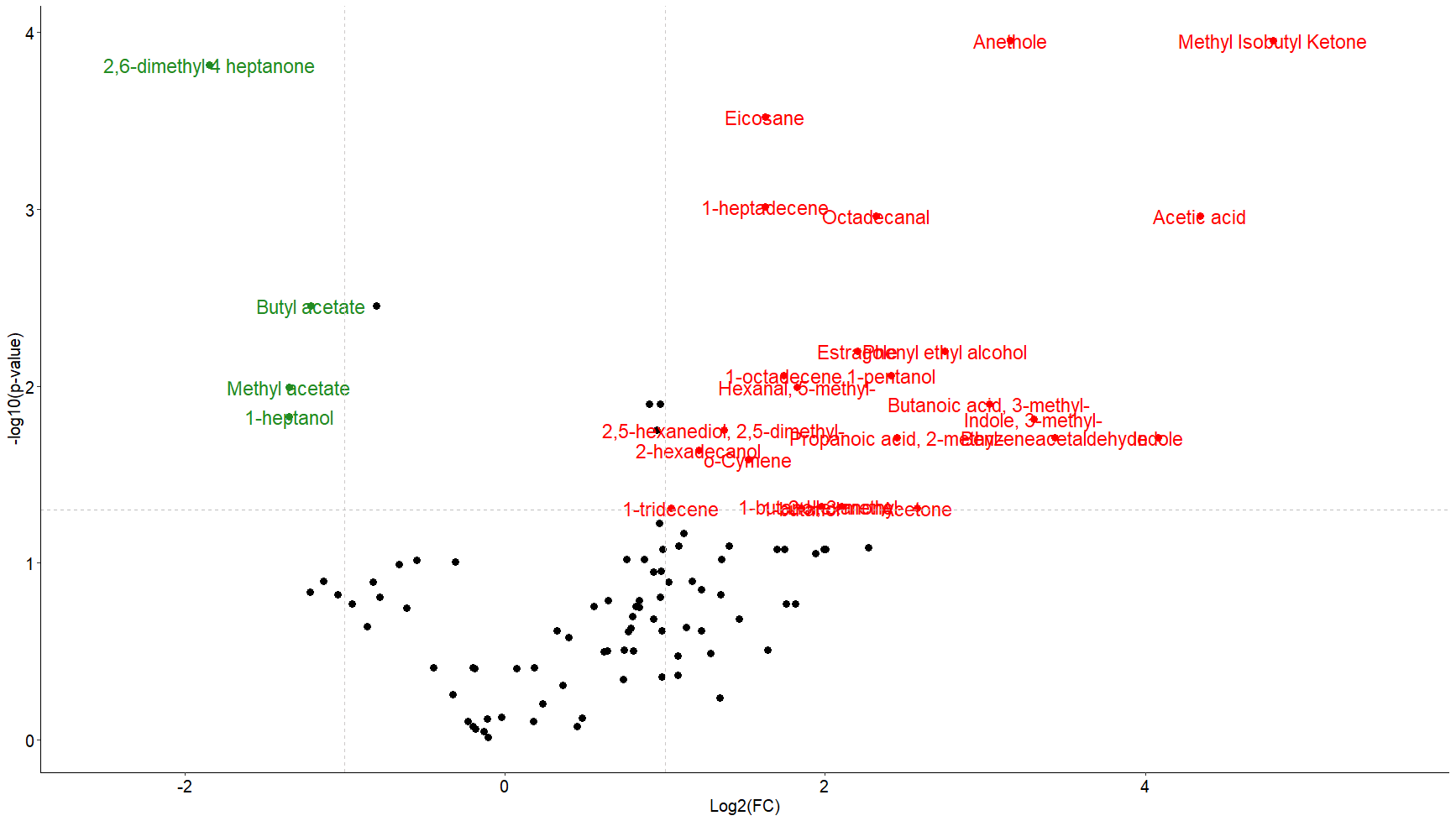
****Supplementary Figure 4B**. **Volcano plot reporting over- and under-expressed VOCs in ASDs vs. CTRLs.** X axis shows log_2_FC. The Fold change (FC) refers to the ratio of average VOCs concentrations for ASDs and CTRLs, i.e., mean [VOCs_(ASDs)_]/mean [VOCs_(CTRLs)_].The y axis shows the -log_10_ of *p* value adjusted by FDR in Wilcoxon test. The over-expressed VOCs for ASDs are reported in red with a FC≥2, and the under-expressed are reported in green with a FC≤-2.


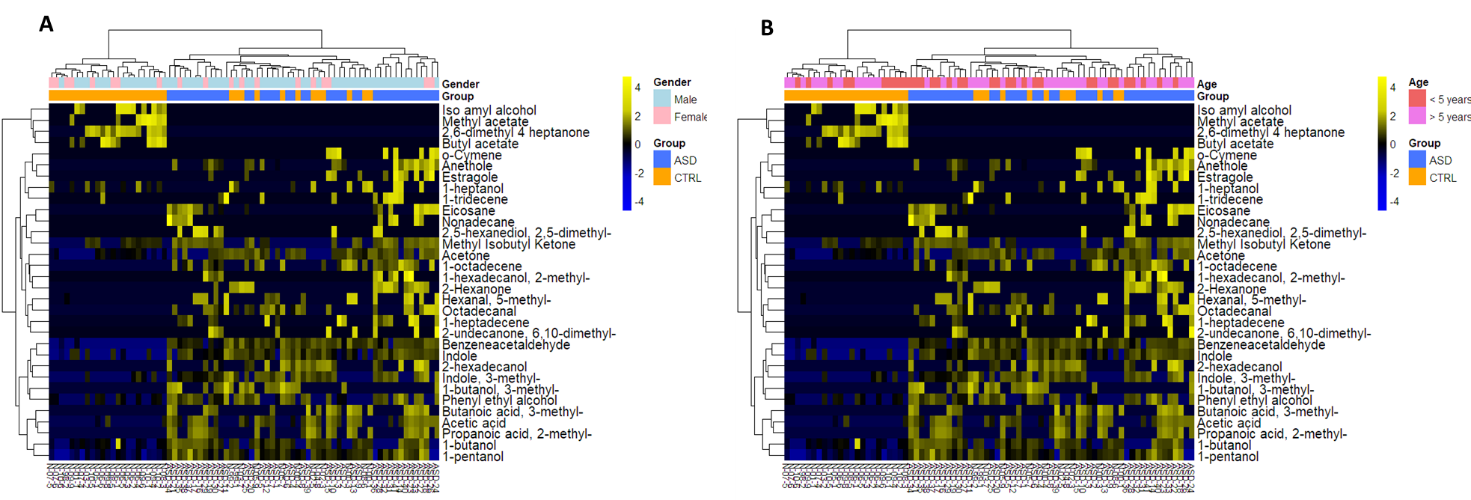


**Supplementary Figure 5**. Heatmap of statistically significant VOCs (p-value ≤ 0.05 corrected with FDR method) between ASDs and CTRLs by considering age (**Panel A**) and gender (**Panel B**). The cluster was constructed using Euclidean distance and Ward's clustering method. The color scale characterizes the Z- score values for each variable: yellow, high level; blue, low level. The column bar colors represent the subject condition category: blue, ASD; orange, CTRLs; light blue, Male; light pink*,* Female; red, subjects <5 years; pink, subjects ≥5 years.


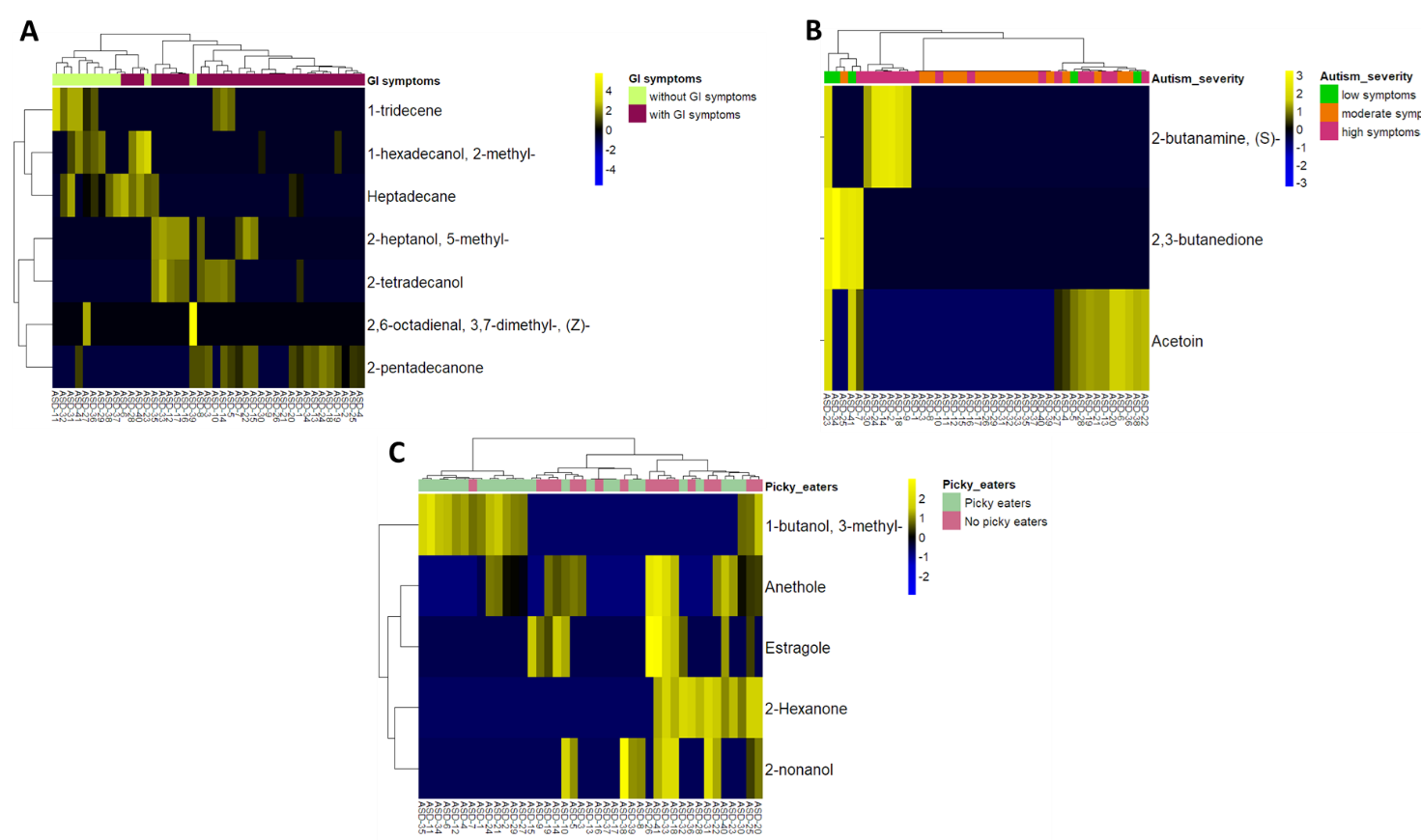


**Supplementary Figure 6**. **Heatmap of statistically significant VOCs (p-value ≤ 0.05 corrected with FDR method) amongst ASDs by considering presence/absence of GI symptoms (Panel A), autism severity symptoms (Panel B) and picky eaters/no picky eaters (Panel C).** The cluster was constructed using Euclidean distance and Ward's clustering method. The color scale characterizes the Z- score values for each variable: yellow, high level; blue, low level. The column bar colors represent the subject condition category: light lime, ASD without GI symptoms; amaranth, ASD with GI symptoms; green, ASD with low symptoms; orange*,* ASD with moderate symptoms; cyclamen, ASD with high symptoms; light green, ASD picky eaters; light cyclamen, ASD no picky eaters.


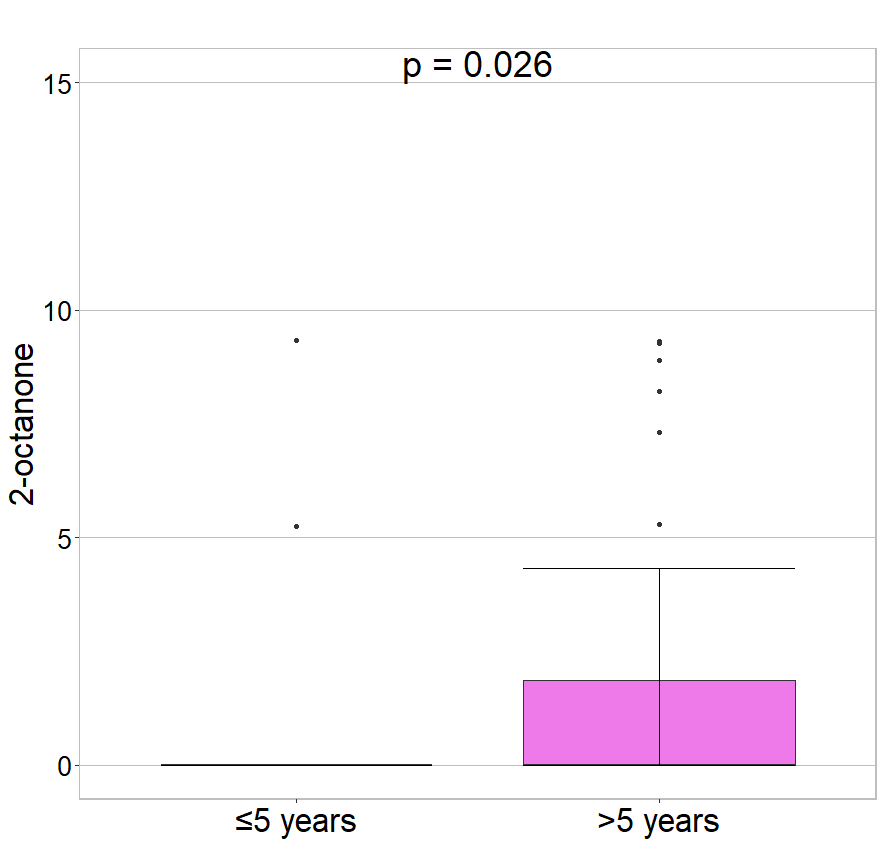

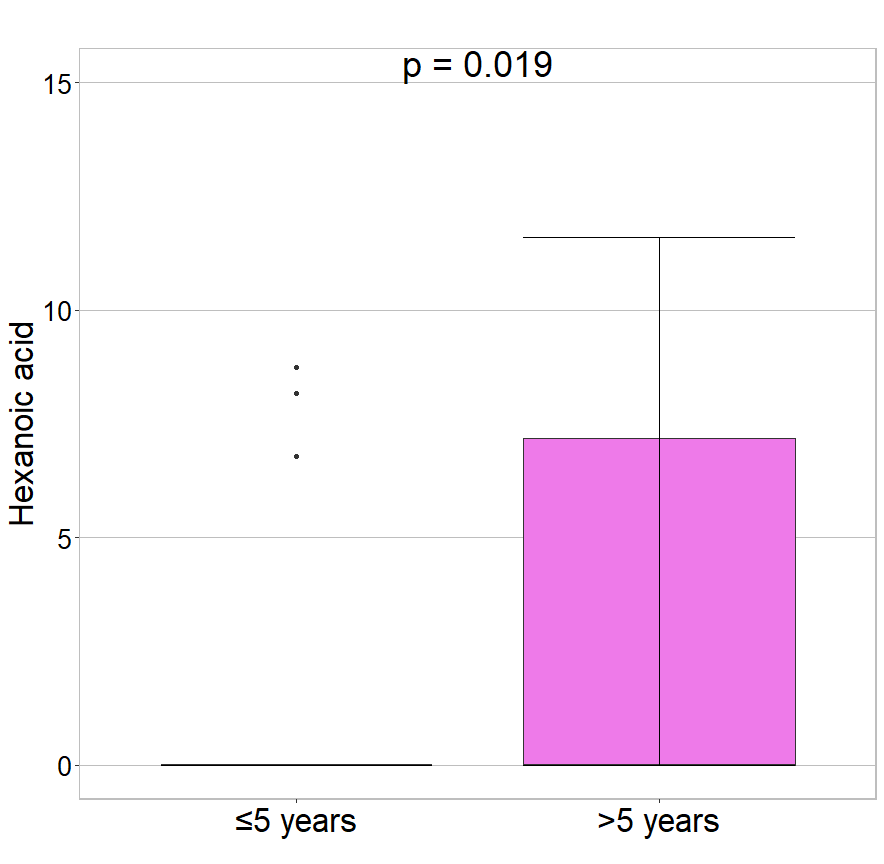

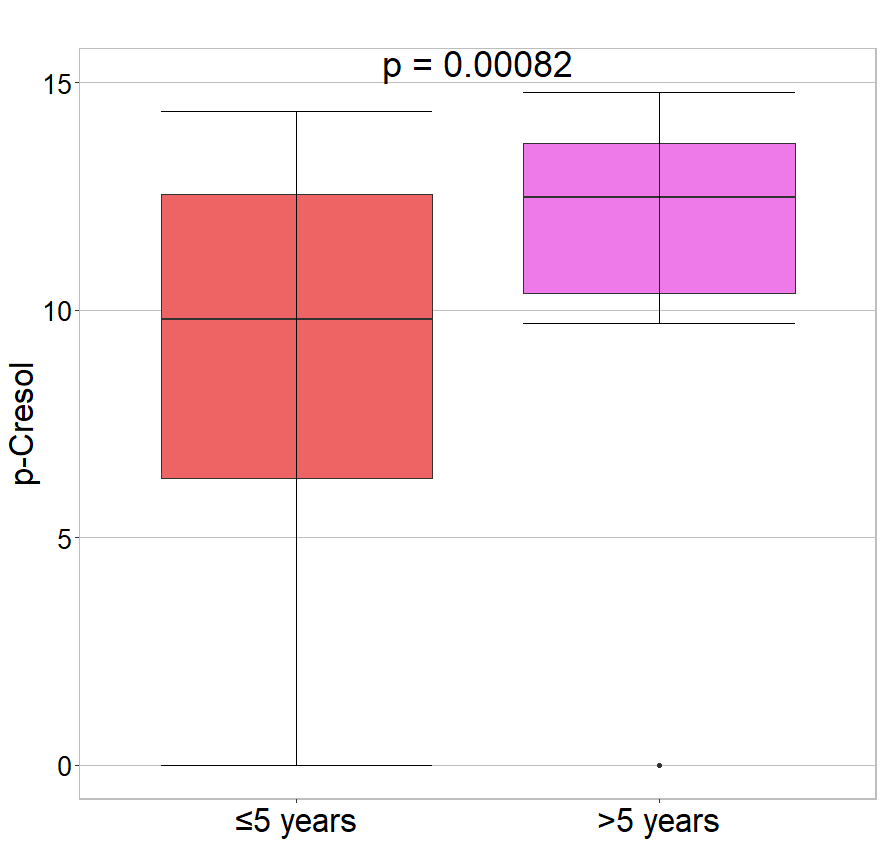

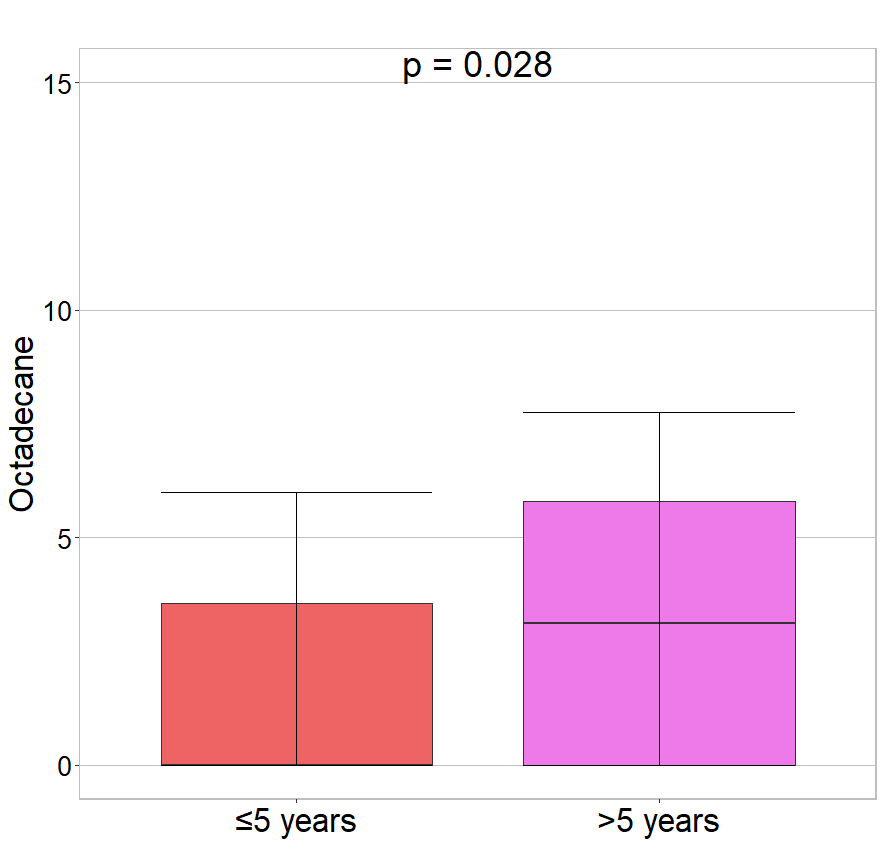

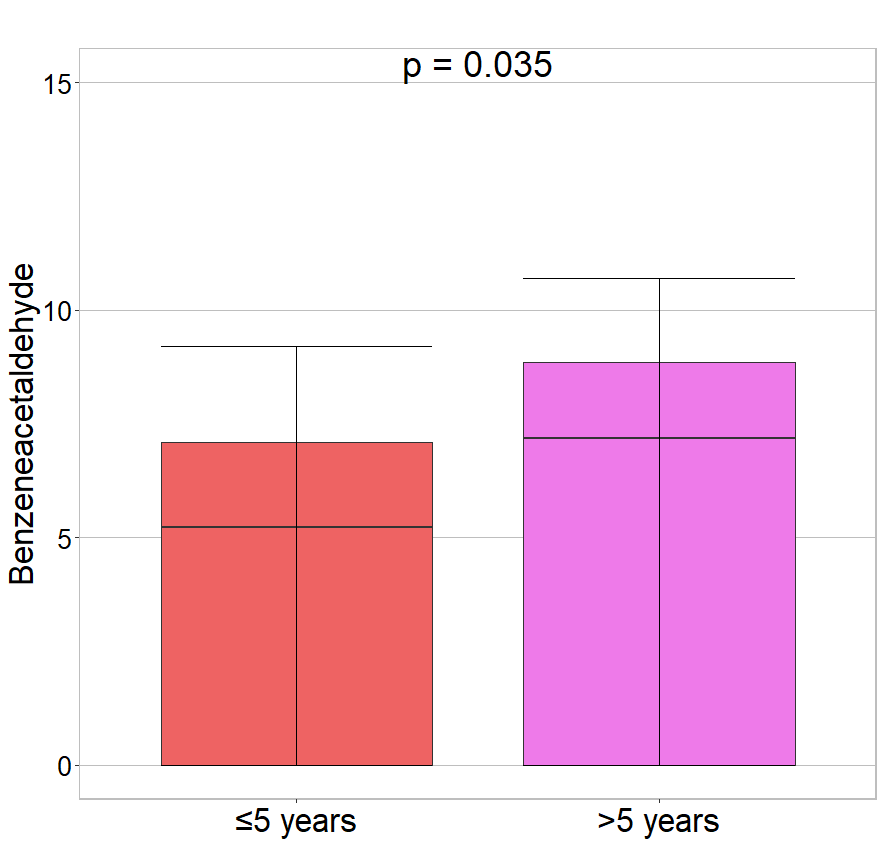

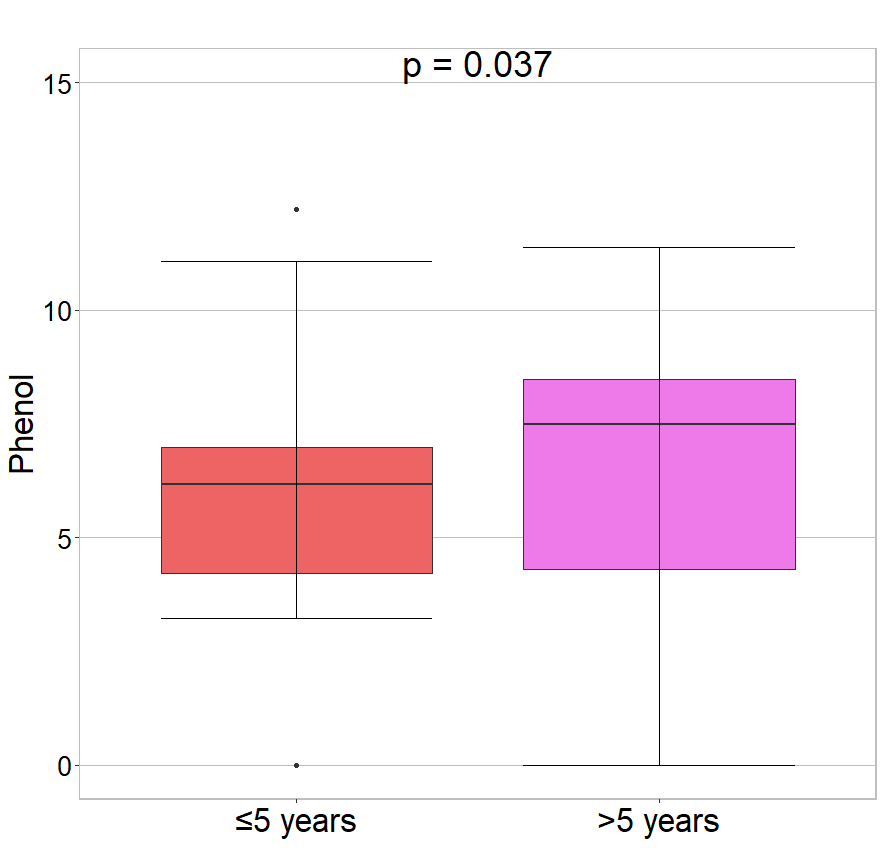

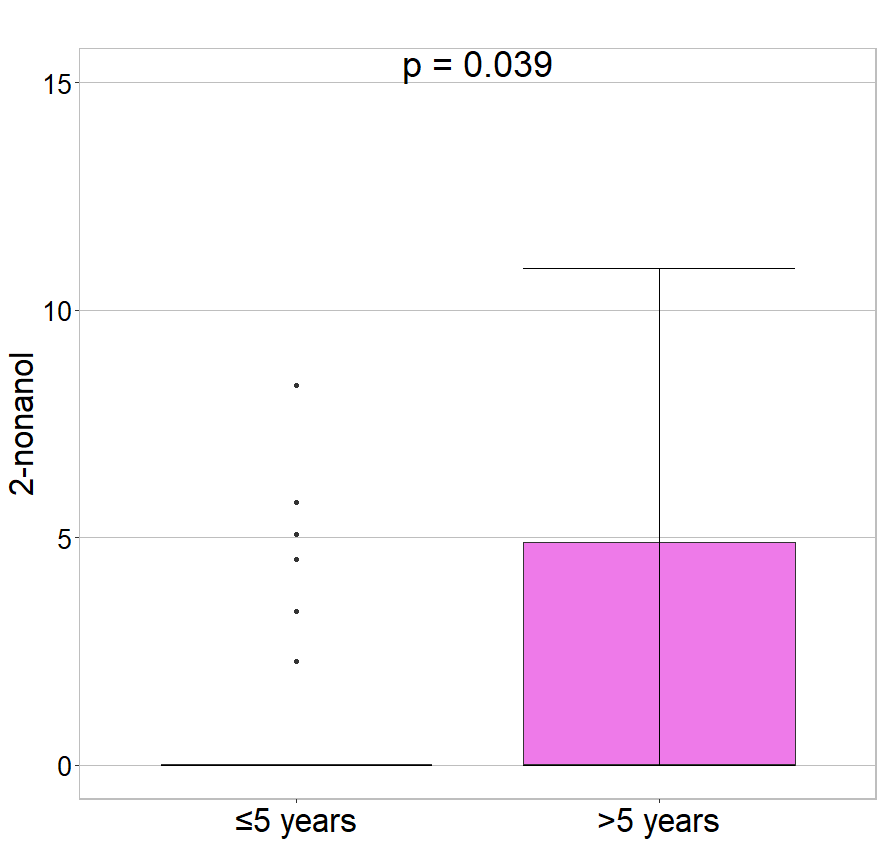

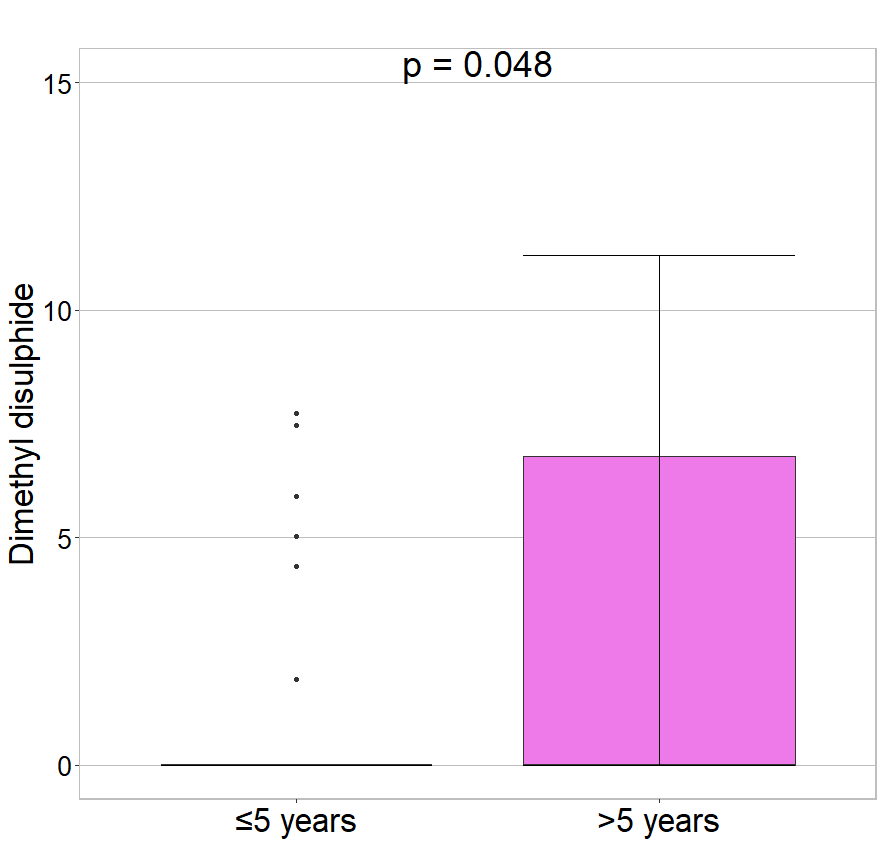

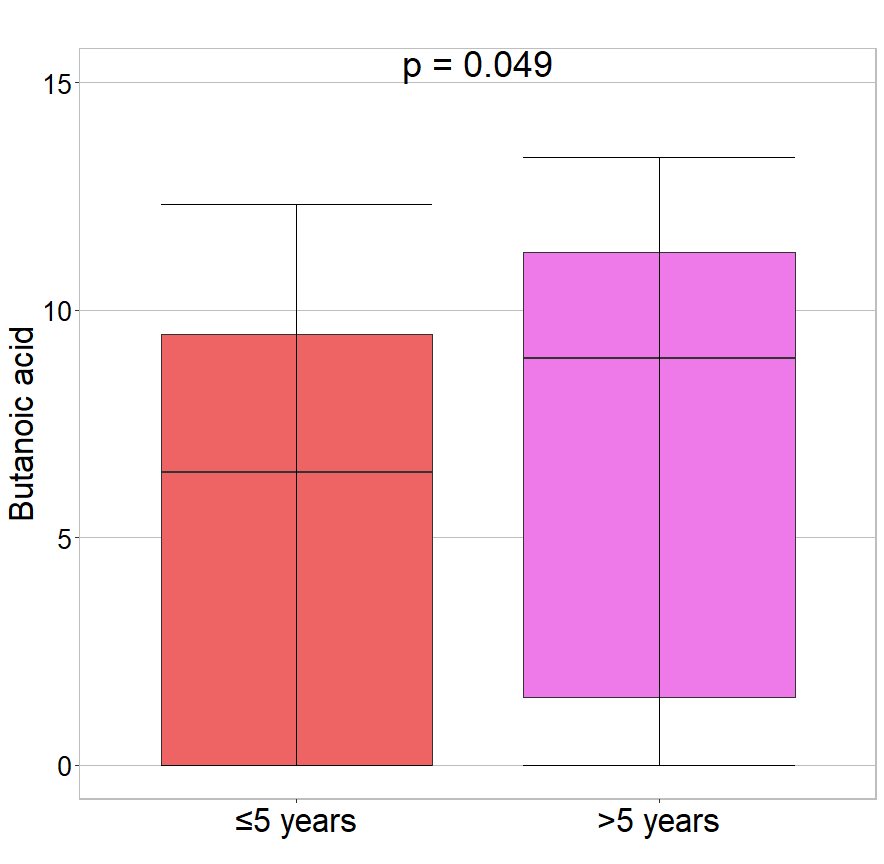


**Su**

**Supplementary Figure 7. Differentially expressed VOCs (*p* value ≤ 0.05, Mann-Whitney test) for ASDs grouped for age: ≤5 years *vs* > 5 years**. Red histograms refer to age ≤5 years; violet histograms refer to age >5 years.


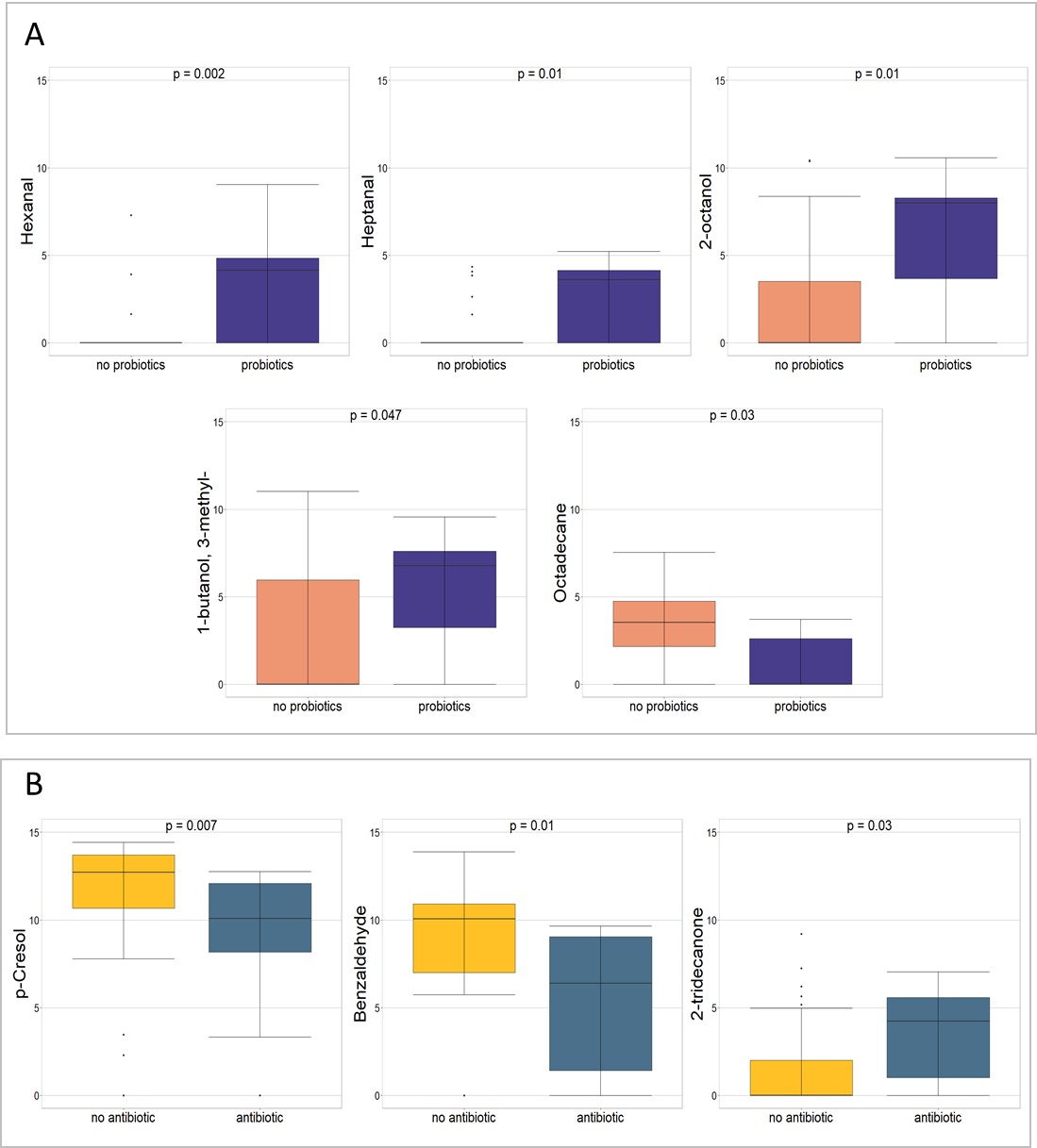


**Supplementary Figure 8. Differentially expressed VOCs (*p* value ≤ 0.05, Mann-Whitney test) for ASDs subgrouped for probiotics’ supplementation and antibiotics’ administration, respectively**. **A**) ASDs without probiotics’ supplementation *vs* ASDs with probiotics’ supplementation. Orange histograms refer to ASDs without probiotics’ supplementation; blue histograms refer to ASDs with probiotics’ supplementation. **B**) ASDs without antibiotics’ administration *vs* ASDs with antibiotics’ administration. Yellow histograms refer to ASDs without antibiotics’ administration; light blue histograms refer to ASDs with antibiotics’ administration.


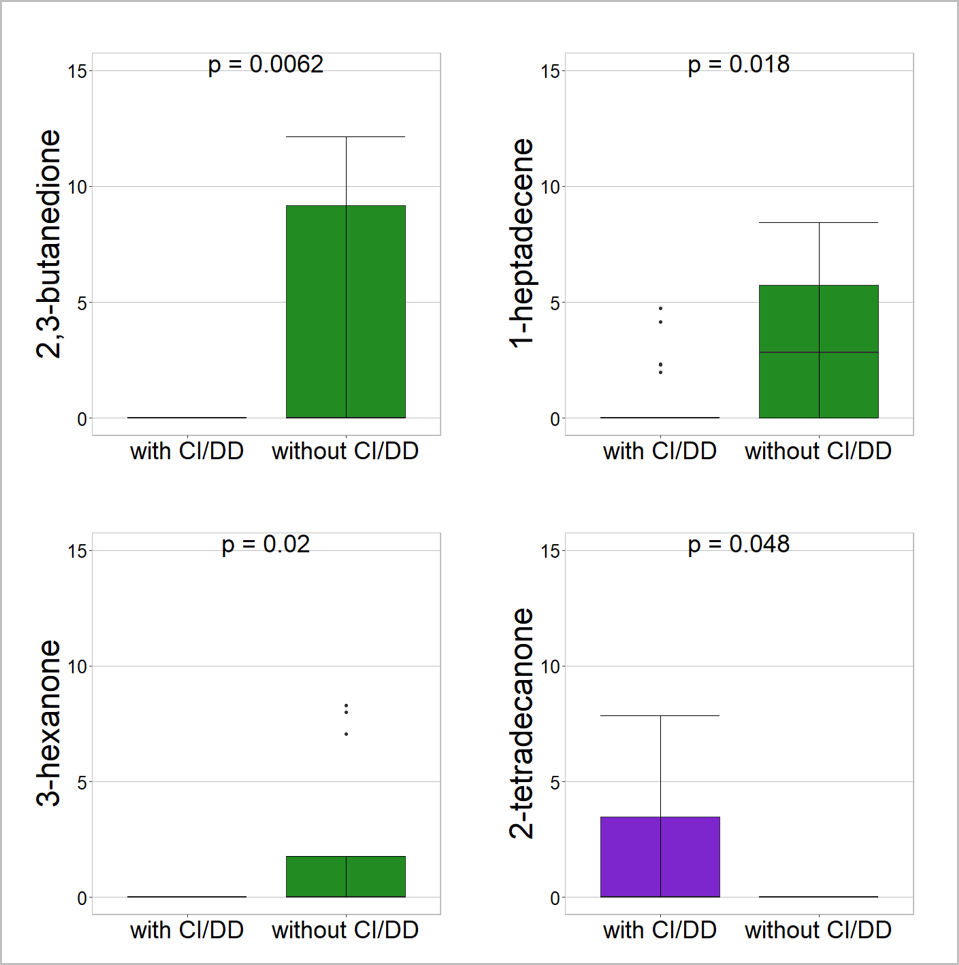


**Supplementary Figure 9. Differentially expressed VOCs (*p* value ≤ 0.05, Mann-Whitney test) for ASDs subgrouped for presence/absence of cognitive impairment/developmental delay.** Green histograms refer to ASDs without cognitive impairment/developmental delay (CI/DD); Violet histograms refer to presence of cognitive impairment/developmental delay (CI/DD).


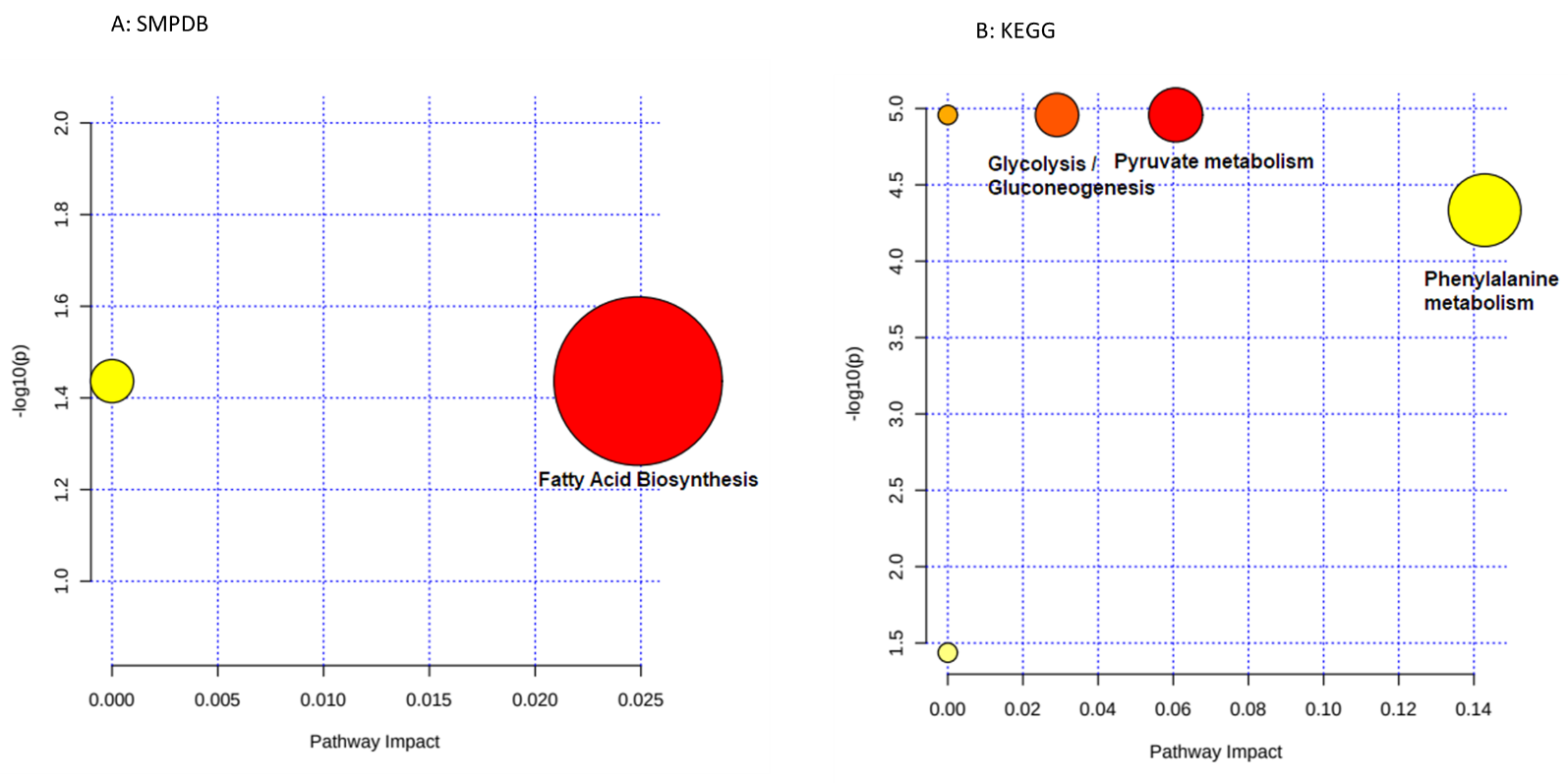


**Supplementary Figure 10. Metabolic Pathway Analysis (MetPA) comparing ASDs *vs* CTRLs.** The − log p-value obtained from the pathway enrichment analysis is plotted on the y-axis, and the pathway impact value derived from the pathway topology analysis is on the x- axis. Each metabolic pathway is represented by a circle; color intensity (yellow to red) is proportional to the p-value, the radius to the pathway impact value. **Panel A**, pathway obtained by using SMPDB database. **Panel B**, pathway obtained by using KEGG database.

**
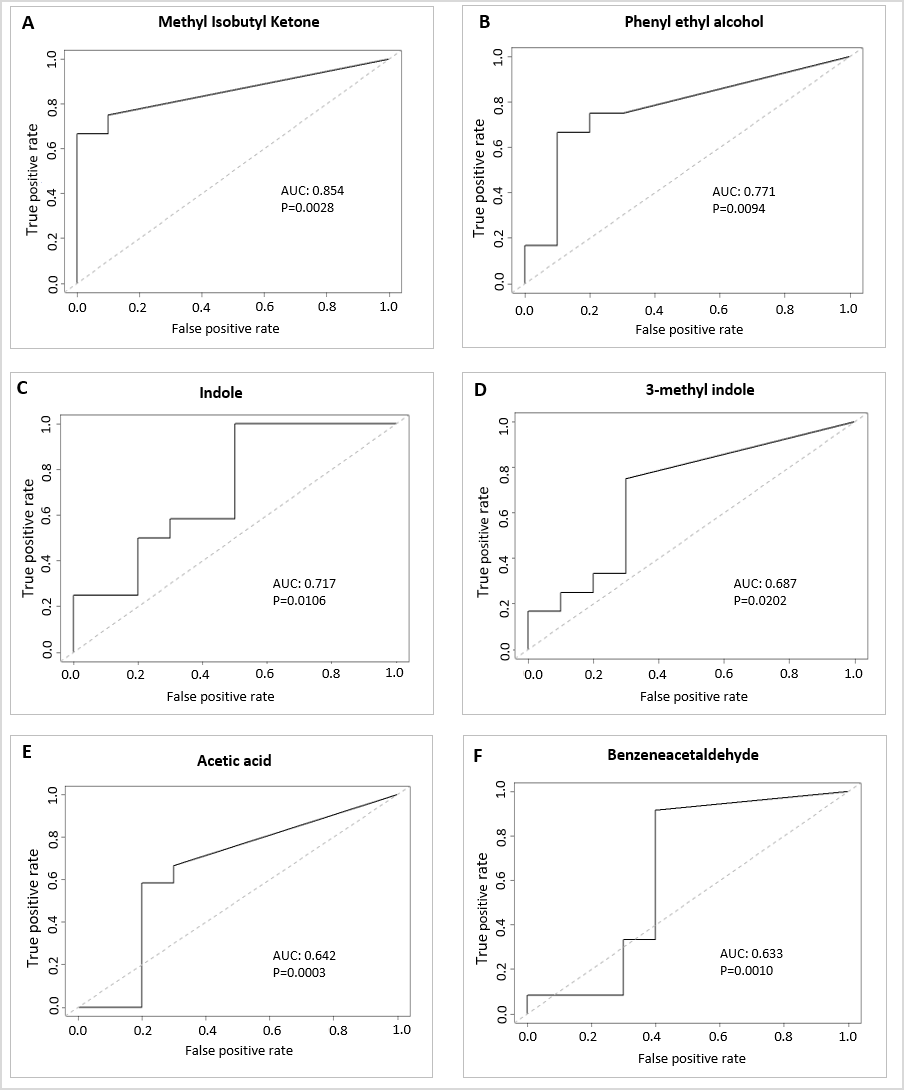
**

**Supplementary Figure 11. Receiver operating characteristic curve (ROC) analysis and logistic regression of 6 VOCs statistically significant associated (*p* ≤0.05) with ASD condition**. AUC; area under the curve.
